# Genome sequencing of the *Trichoderma reesei* QM9136 mutant identifies a truncation of the transcriptional regulator XYR1 as the cause for its cellulase-negative phenotype

**DOI:** 10.1186/s12864-015-1526-0

**Published:** 2015-04-20

**Authors:** Alexander Lichius, Frédérique Bidard, Franziska Buchholz, Stéphane Le Crom, Joel Martin, Wendy Schackwitz, Tina Austerlitz, Igor V Grigoriev, Scott E Baker, Antoine Margeot, Bernhard Seiboth, Christian P Kubicek

**Affiliations:** Research Division Biotechnology and Microbiology, Institute of Chemical Engineering, Vienna University of Technology, A-1060 Vienna, Austria; IFP Energies Nouvelles, 1-4 Avenue de Bois-Préau, 92852 Rueil-Malmaison, France; Sorbonne Universités, UPMC Université Paris 06, Institut de Biologie Paris Seine (IBPS), FR 3631, Département des Plateforme, F-75005 Paris, France; US Department of Energy, Joint Genome Institute, 2800 Mitchell Avenue, Walnut Creek, CA 94598 USA; Environmental Molecular Sciences Laboratory, Pacific Northwest National Laboratory, Richland, WA 99354 USA

**Keywords:** Single nucleotide polymorphism, SNP, Indel, Comparative genomics, Classical mutant, XYR1, Transcription factor shuttling, Cellulases, *Trichoderma reesei*, QM9136

## Abstract

**Background:**

*Trichoderma reesei* is the main industrial source of cellulases and hemicellulases required for the hydrolysis of biomass to simple sugars, which can then be used in the production of biofuels and biorefineries. The highly productive strains in use today were generated by classical mutagenesis. As byproducts of this procedure, mutants were generated that turned out to be unable to produce cellulases. In order to identify the mutations responsible for this inability, we sequenced the genome of one of these strains, QM9136, and compared it to that of its progenitor *T. reesei* QM6a.

**Results:**

In QM9136, we detected a surprisingly low number of mutagenic events in the promoter and coding regions of genes, *i.e.* only eight indels and six single nucleotide variants. One of these indels led to a frame-shift in the Zn_2_Cys_6_ transcription factor XYR1, the general regulator of cellulase and xylanase expression, and resulted in its C-terminal truncation by 140 amino acids. Retransformation of strain QM9136 with the wild-type *xyr1* allele fully recovered the ability to produce cellulases, and is thus the reason for the cellulase-negative phenotype. Introduction of an engineered *xyr1* allele containing the truncating point mutation into the moderate producer *T. reesei* QM9414 rendered this strain also cellulase-negative. The correspondingly truncated XYR1 protein was still able to enter the nucleus, but failed to be expressed over the basal constitutive level.

**Conclusion:**

The missing 140 C-terminal amino acids of XYR1 are therefore responsible for its previously observed auto-regulation which is essential for cellulases to be expressed. Our data present a working example of the use of genome sequencing leading to a functional explanation of the QM9136 cellulase-negative phenotype.

**Electronic supplementary material:**

The online version of this article (doi:10.1186/s12864-015-1526-0) contains supplementary material, which is available to authorized users.

## Background

Public concerns about the consequences of the continued production of fuels and chemicals from fossil carbon sources have today led to intensive attempts to switch to the use of renewable carbon resources for future energy supply, such as lignocellulosic biomass from agricultural crop residues, grasses, wood and municipal solid waste. This affects not only the production of ethanol as a biofuel but also a range of key biochemical building blocks for biorefineries, including 1,4-dicarboxy acids, 3-hydroxypropionic acid, levulinic acid, and polyols; to only name a few. Thereby, the costs of cellulases and hemicellulases needed for hydrolysis of the plant biomass to soluble substrates contribute substantially to the price, and much cheaper sources of these enzymes are urgently required [1]. Consequently, there has been a renaissance in studies aimed at understanding and improving cellulase efficiency and productivity.

**Figure 1:**
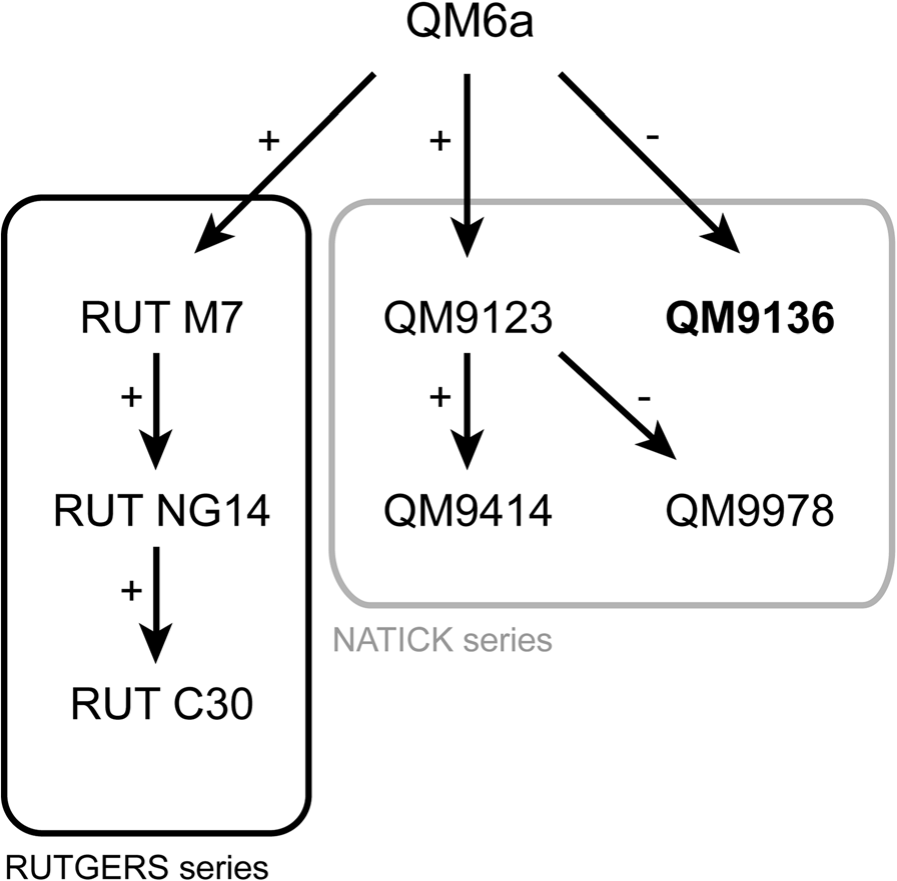
Pedigree of the cellulase-negative strain QM9136. UV light and chemical mutagenesis programs of the NATICK [3,4,9] and RUTGERS [27] series lead to the selection of *T. reesei* strains with improved (+) or impaired (−) cellulase production. Adapted with permission from [2].

*Trichoderma reesei* (teleomorph *Hypocrea jecorina*), a fungus commonly found on decaying wood within a small latitude North and South of the equator, is a model fungus for cellulase and hemicellulase production [2]. Unlike other industrial fungi, mutants were all derived from a single wild-type isolate (QM6a) which was originally found during WWII on the Solomon Islands because of its ability to destroy cotton fabrics of the US Army [3]. During the first oil crisis in the 1970s, *T. reesei* QM6a was found being the best cellulase producer in a large screen of tropical fungi contained in the US Army Quatermaster (QM) culture collection [4]. Random mutagenesis was then used to improve this strain and establish industrial production. In the attempt to understand the mechanisms of improvement in these strains, the genomes of some strains of these mutagenesis lineages (*i.e.* QM9123, QM9414; NG14, RUT C30; see Figure 1) have recently been sequenced [5-8]. This led to the identification of a high number of mutated alleles, their causal involvement in enhanced cellulase production, however, still awaits verification [2].

In the course of the earliest attempts to improve *T. reesei* QM6a by mutagenesis, the mutant strain QM9136 was obtained that was unable to grow on cellulose and that did not form any cellulases on cellulose or any other inducer, such as lactose or sophorose, but otherwise displayed a normal phenotype [9-11]. The genetics underlying this cellulase-negative phenotype of this mutant are essentially unknown.

Here we report the sequencing of the genome of the cellulase-negative *T. reesei* strain QM9136. We show that – among a few other mutations that likely are irrelevant to cellulase formation – this strain contains a frame-shift mutation in the major cellulase regulator gene *xyr1* [10], and that its cellulase production can be fully rescued by reintroduction of the wild-type *xyr1* at its locus. We further show that this frame-shift mutation causes a 140 C-terminal amino acid truncation of XYR1, which interferes with the positive auto-regulatory feedback mechanism required for *de novo* XYR1 biosynthesis, efficient nuclear accumulation and transcriptional activity towards cellulase and hemicellulase gene expression.

## Results

### Identification of mutations in QM9136

The strain *T. reesei* QM9136 has been isolated by a single round of X-ray mutagenesis using a linear particle accelerator from the wild type strain QM6a [9], and was identified to be unable to produce cellulases [9,10]. Phenotypically, colony growth of *T. reesei* QM9136 on agar plates supplemented with either D-glucose, cellobiose or lactose was comparable to that of its parent *T. reesei* QM6a and the moderate cellulase producer QM9414 (Additional file 1: Figure S1). Interestingly, the ability of QM9136 to grow on cellulose is limited to cultivation on agar plates, and does not occur when the strain is grown in liquid culture. This phenomenon is not fully understood, but is shared with other cellulase deficient strains, including ∆*xyr1* [12]. Nevertheless, in order to understand the origin of the cellulase-negative phenotype, we conducted whole genome comparisons between the natural isolate, the QM6a strain, and the mutant strain QM9136 which we sequenced using high-throughput Illumina sequencing. To get the complete picture of genetic modifications in that strain, this analysis included identification of the SNVs (Single Nucleotide Variants), InDels (small Insertions and Deletions) and large structural variations (deletion, duplication, translocation, insertion). The final list of mutations identified in the strain QM9136 (Table 1) has been established from *in silico* analysis followed by an expert analysis (see Methods). In total, 14 mutations have been detected: eight InDels, six SNVs and no structural variations. Compared to the other mutant strains sequenced [5-7], the number of mutations is much lower than that in *T. reesei* NG14 and RUT C30, but in the same order as in QM9123 and QM9414. This could be due to the fact that strains NG14 and RUT C30 were selected not only for cellulase hyperproduction but also for resistance against metabolic inhibitors [5,6].

**Table 1:** **Mutations found in** ***T. reesei*** **QM9136 when compared with its parent** ***T. reesei*** **QM6a**

**SNP_id**	**Mutation**	**Mutation effect**	Position* [nt]	**Transcript**	**Element**	**Annotation**
4_329479	SNV	G > C		No		
4_1496656	SNV	A > T (codon : TTC > TAC; aa : F > Y)	+38	Trire2:46238	exon	putative S-adenosylmethionine synthetase SAM1
11_1146347	SNV	C > G		No		
18_608684	SNV	C > G (codon : AGT > ACT; aa : S > T)	+71	Trire2:50707	exon	putative cell-cycle regulated activator of the anaphase-promoting complex/cyclosome CDC1
19_523375	SNV	A > G		No		
31_173856	SNV	C > G (codon: CCT > GCT; aa : P > A)	+1189	Trire2:124043	exon	putative glycoside hydrolase family 18 CHI18-14
1_518722	InDel	−1:A	+19	Trire2:102500	exon	MRSP1/expansin-like-orphan protein
4_1479439	InDel	−1:A		No		
5_692881	InDel	+1:C		No		
11_202303	InDel	−1:A	+2294	Trire2:122208	exon	transcription factor XYR1
16_183301	InDel	−1:G		Trire2:109416	promoter	putative protein of unknown function
18_234489	InDel	−1:A		No		
19_227418	InDel	−8:GCCCGGCG	+19	Trire2:66687	exon	β-1,4-mannosyl-glycoprotein
						β-1,4-N-acetylglucosaminyltransferase (EC 2.4.1.144
23_109645	InDel	−1:T		Trire2:5387	promoter	putative NADH-ubiquinone oxidoreductase

*in the spliced transcript.

In order to establish the list of genes impacted by these mutations, we systematically scanned for genes in an 800 bp window flanking each mutation. From the initial list of 14 mutations, eight genes were impacted either in their exons, their introns, or within 800 bp upstream or 200 bp downstream of the coding region (CDR). They were located on eight different scaffolds, and thus not clustered. Nevertheless, it was intriguing to note that they were all located either in the 5′ or the 3′ fifth of the scaffold (Additional file 2: Table S1). In addition, all mutations occurred in a genomic region that is syntenic across *T. reesei, T. virens* and *T. atroviride* (C.P. Kubicek, unpublished data).

In silico *analysis of the mutations in* T. reesei *QM9136 identifies a truncation in the cellulase regulator XYR1*.

Three SNVs were present in the CDR of the following genes: the S-adenosylmethionine synthase SAM1 (Trire2:46238); a WD-repeat containing protein that has high similarity to the cell cycle regulator CDC1 (Trire2:50707), and the subgroup B chitinase CHI18-14 (Trire2:124043; [13]; Table 1). However, in the former two proteins these SNVs only resulted in conserved amino acid changes (F13Y and S24T, respectively). The change in CHI18-14 (P389A) occurs within the C-terminal CBM1-type cellulose binding domain. However, this P is not conserved, and is substituted by G or C in CBM1 orthologues from other fungi [14]. We therefore consider it unlikely that this change significantly alters the functional properties of CHI18-14.

The five indels had a more dramatic effect: in the expansin-like protein Trire2:102500 and the GT17 β-1,4-mannosyl-glycoprotein 4-β-*N*-acetylglucosaminyltransferase (Trire2:66687), each of the two single nucleotide losses occurred proximal to the N-terminus, and gave rise to a completely different or truncated protein sequence, respectively (Table 1). As for the gene encoding the 24 kDa subunit of the NADH-ubiquinone oxidoreductase (Trire2:5387), and a protein of unknown function (Trire2: 109416), the loss of a T and a G, respectively, occurs in their promoters and the consequences of this change cannot be predicted.

### Consequences of the truncation on XYR1 structure

The most interesting mutation, however, occurred in the gene encoding the transcriptional regulator XYR1 (Trire2:122208). The deletion of A2294 in the spliced transcript leads to a frame-shift from T2295 onwards that still encodes 16 amino acids (L765-F780: LSSTSSLRISGIPSTF) that are different to those in the native XYR1, before the first premature amber stop codon eventually terminates translation (Additional file 3: Figure S2). Consequently, 140 C-terminal amino acids are absent resulting in the truncated XYR1^1–780^ protein.

**Figure 2:**
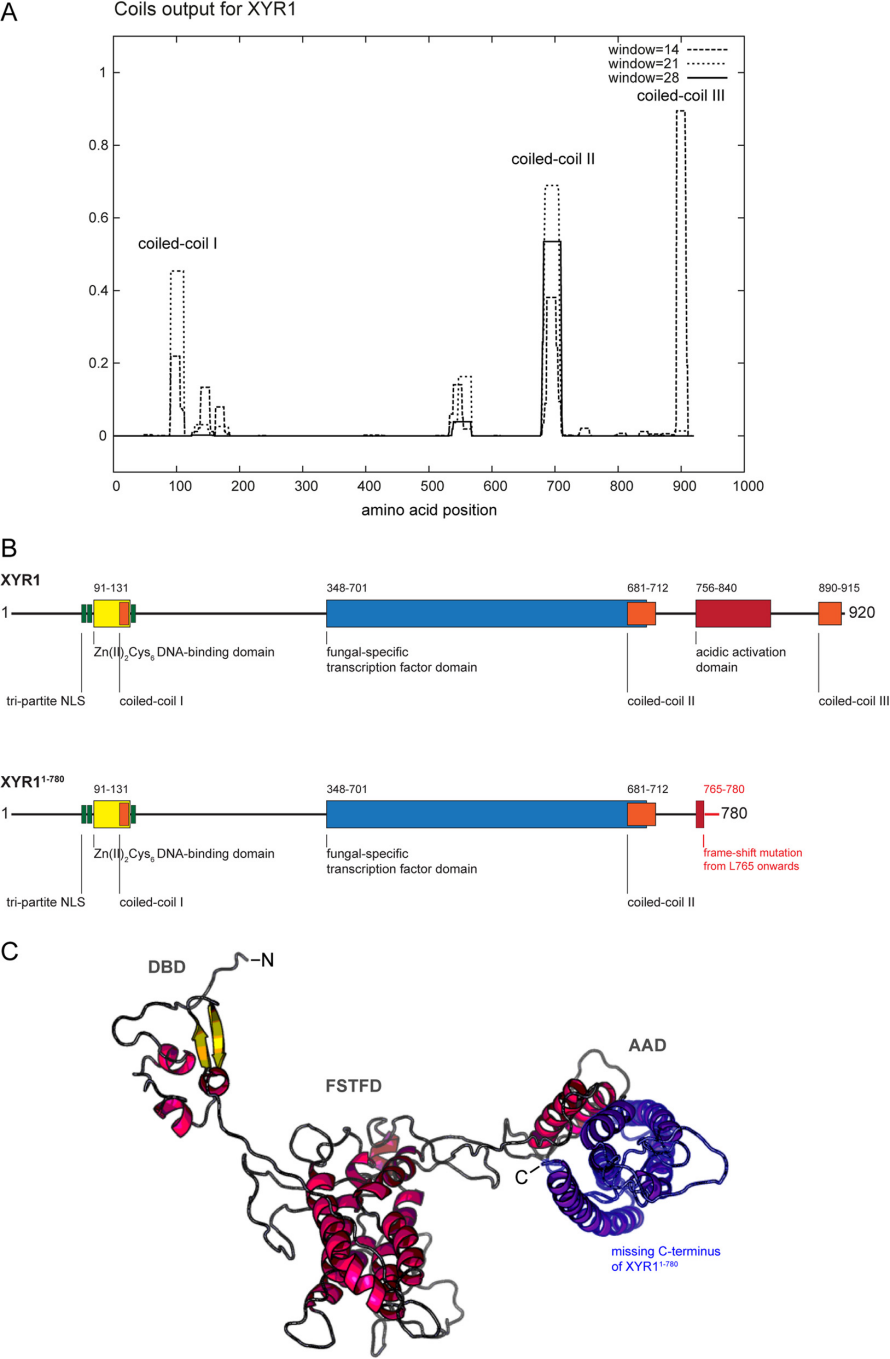
XYR1 structure and structural consequences of the truncated XYR^1–780^. **(A)** Predicted coiled-coil domains in XYR1 using COILS, which compares a sequence to a database of known parallel two-stranded coiled-coils and derives a similarity score from which the probability that the sequence will adopt a coiled-coil confirmation is calculated. **(B)** XYR1 contains a classical tri-partite nuclear localisation signal (NLS; green) which directs the transcription factor into the nucleus. XYR1’s DNA-binding domain (DBD; yellow) is orthologous to that of Gal4 from *Saccharomyces cerevisiae*. The first coiled-coil domain of XYR1 (orange) is located at the end of the DBD. The filamentous fungal-specific transcription factor domain (FSTFD; blue) regulates target gene expression and constitutes the biggest part of the protein. A second coiled-coil domain (orange) overlaps with the C-terminal end of the FSTFD. The acidic activation domain (AAD; dark red) is a characteristic feature of Zn_2_Cys_6_-type transcription factors. The third coiled-coil domain (orange) sits at the very C-terminus, and likely mediates homodimerisation of XYR1. Hence, the truncated XYR1^1–780^ protein encoded in QM9136 almost completely lacks the AAD and fully lacks the third coiled-coil domain due to a frame-shift mutation from L765 onwards. The terminal 16 amino acids (L765-F780; light red) encode a non-sense sequence that finds no BLASTp hit in the *T. reesei* genome. **(C)** 3D-reconstruction of XYR1. RaptorX protein structure prediction software (raptorx.uchicago.edu) identifies three functional domains in XYR1, resembling DNA-binding domain (DBD), fungal-specific transcription factor domain (FSTFD) and acidic-activation domain (AAD), perfectly matching our manual annotation **(B)**. Five of the seven α-helices comprising the AAD are missing in the truncated XYR1^1–780^ protein (highlighted in blue). The closest structural orthologs are the Zn_2_Cys_6_-type transcriptional regulators Ppr1 and Gal4 from *S. cerevisiae*, both known to be active as non-symmetrical homodimers [63].

A Pfam domain search [15] identified two major functional domains in XYR1: the N-terminal Zn_2_Cys_6_-cluster involved in DNA binding (Gal4-like DNA-binding domain, DBD; R91-K131; 1.6e^−08^), and a fungal-specific transcription factor domain (FSTFD; R348-E701; 2.2e^−29^). Using COILS [16], three coiled-coil regions were identified: one covering the Zn_2_Cys_6_-cluster and 51 amino acids C-terminal of it (R91-L182), one at the C-terminal end of the FSTFD (S681-P712), and one at the C-terminal end of XYR1 (R890-G915) (Figure 2A). This indicates that the truncation in QM9136 starts after the FSTFD. The missing amino acids have a composition with a bias towards acidic amino acids resulting in an isoelectric point of 4. This domain thus likely resembles the acidic activation domain (AAD) that is an invariant part of Zn_2_Cys_6_ transcription factors [17]. An alignment of XYR1 orthologues from several Pezizomycota shows that the missing C-terminus of XYR1^1–780^ is otherwise highly conserved in all fungi (Additional file 4: Figure S3). This suggests that these 140 C-terminal amino acids are important for the function of this protein. Figure 2B shows a schematic domain comparison of full-length XYR1 and truncated XYR1^1–780^. Template-based protein structure modelling of this acidic activation domain showed that it forms a bundle of helices of which the last four constitute the above mentioned coiled-coil domain (Figure 2C).

The overall protein stability (estimated by computing the instability index by PROTPARAM; [18]) was determined to be 54.21 and 54.45 for XYR1 and XYR1^1–780^, respectively. While this suggests that the truncation does not impact structural stability compared to the full-length protein, it should be noted that both the native and the mutant XYR1 are classified as unstable, indicating a generally high protein turn-over rate as part of XYR1's cellular function.

### Introduction of native *xyr1* into *T. reesei* QM9136 rescues the mutant growth phenotype

**Figure 3:**
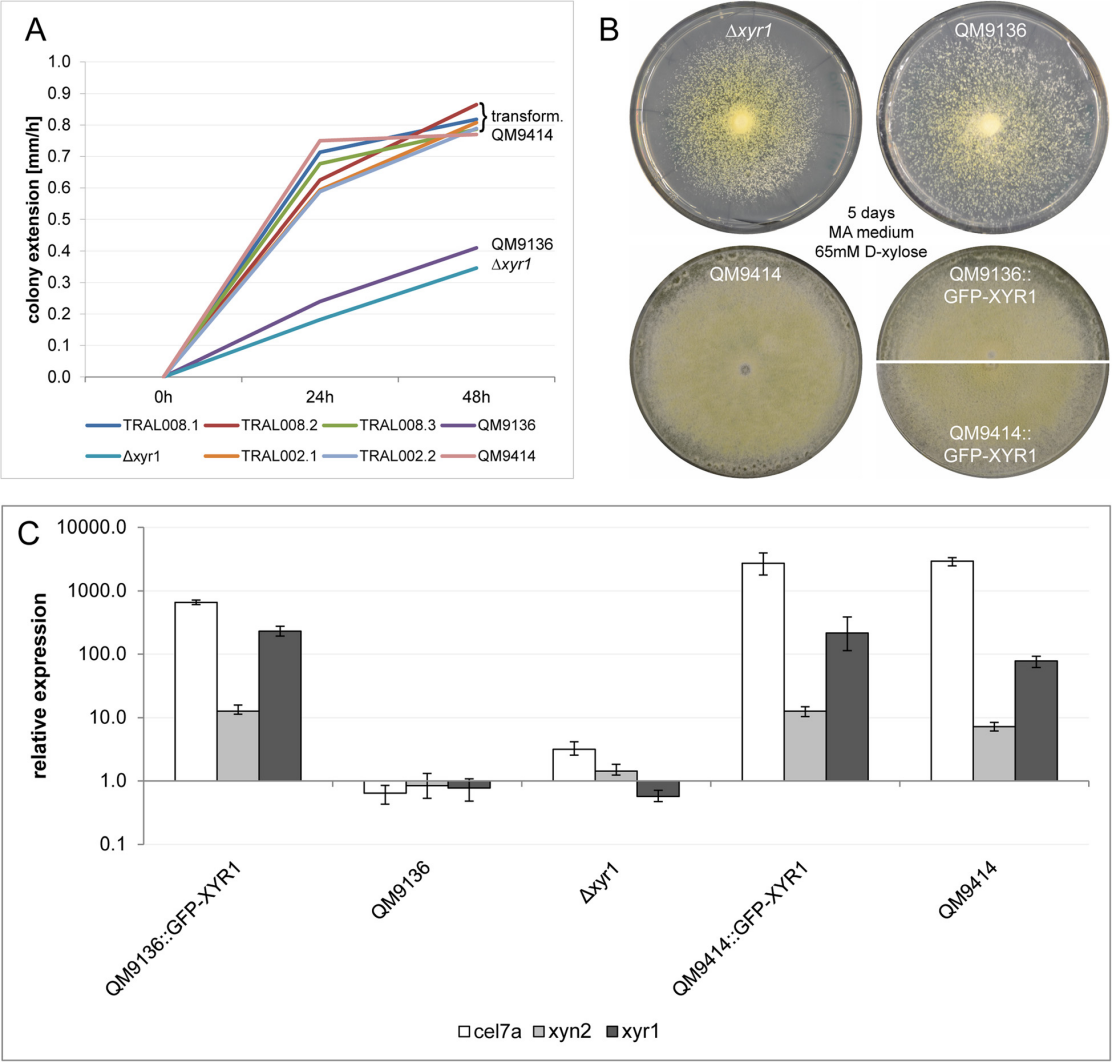
Gene replacement with full-length GFP-XYR1 construct rescues XYR1-loss-of-function phenotype of QM9136. **(A)** QM9136::GFP-XYR1 transformants (TRAL008 clones 1 to 3) grow on 65 mM D-xylose show colony extension speeds comparable to the moderate producer strain QM9414 and QM9414::GFP-XYR1 transformants (TRAL002 clones 1 and 2). QM9136 and ∆*xyr1* are sensitive to 65 mM D-xylose and display significantly reduced colony extension speeds. **(B)** Macroscopic colony phenotypes of GFP-XYR1 transformants TRAL002 and TRAL008, and control strains after 5 days on MA medium supplemented with 65 mM D-xylose. The XYR1-loss-of-function strains ∆*xyr1* and QM9136 develop a slower and less dense growing mycelium, however, start conidiation earlier, compared to the reference strain QM9414. Expression of GFP-XYR1 restores wild type morphology in QM9136 but does not alter QM9414. **(C)** Gene expression of the major cellulase *cel7a*, xylanase *xyn2* and transcription factor *xyr1* itself, induced by one hour exposure to 1.4 mM sophorose, are restored in QM9136::GFP-XYR1 transformants (TRAL008). The XYR1-loss-of-function strains QM9136 and ∆*xyr1* show now transcriptional response to induction by sophorose. Both cellulase-positive controls, QM9414 and QM9414::GFP-XYR1 transformants (TRAL002) respond as expected with upregulated *cel7a*, *xyn2* and *xyr1* expression. Error bars show standard deviations (n = 6 from three experimental and two biological replicates).

The identification of a mutation in QM9136 that truncates the XYR1 cellulase regulator protein led us to assume that this may be the major or exclusive mutation that gave rise to the cellulase-negative phenotype. In order to test this, we replaced the mutated *xyr1* locus in *T. reesei* QM9316 with a construct comprising a 2.9 kb wild-type copy of *xyr1* N-terminally linked to 717 bps encoding for green fluorescent protein (*gfp*), and flanked by 1025 and 800 bps of its 5′ and 3′ UTRs, respectively. We then grew all strains on 65 mM D-xylose as sole carbon source, which results in a compact colony phenotype in case XYR1 function is compromised. As shown in Figure 3A and B, transformation with the *gfp-xyr1* fusion gene fully restored the typical growth defective phenotype of XYR1 loss-of-function mutants on 65 mM D-xylose [19] and matched the average colony extension speed as well as macroscopical morphology of *T. reesei* QM9414 and QM9414 transformed with the identical GFP-XYR1 construct (TRAL002: QM9414∆t*ku70*, P*xyr1*::*gfp-xyr1*::T*xyr1*).

To test whether nuclear import of GFP-XYR1 in a QM9136 background is also sufficient to restore cellulase and hemicellulase gene expression, we performed correlated gene expression analysis on mycelia from liquid cultures induced by 1.4 mM sophorose. The rescued QM9136 transformants indeed, expressed the major cellulase cellobiohydrolase I gene *cel7a* (*cbh1*) at levels comparable to strain QM9414 after one hour of induction with sophorose. No cellulase gene expression was observed in QM9136 or the ∆*xyr1* mutant strain cultivated under identical conditions (Figure 3C). Consistent data were obtained for the xylanase II-encoding gene *xyn2*, hence confirming earlier data [10] that QM9136 is also deficient in hemicellulase formation (Figure 3C). While the experimental error inherent in these analyses cannot completely exclude that one of the other mutations detected (Table 1) would contribute to a very minor extent (<5%) to the cellulase-negative phenotype, we conclude that the mutation in *xyr1* has the most significant impact on QM9136′s inability to produce cellulases and hemicellulases.

### A genetically engineered *xyr1*^*∆A2294*^ allele reproduces the QM9136 mutant phenotype in *T. reesei* QM9414

**Figure 4:**
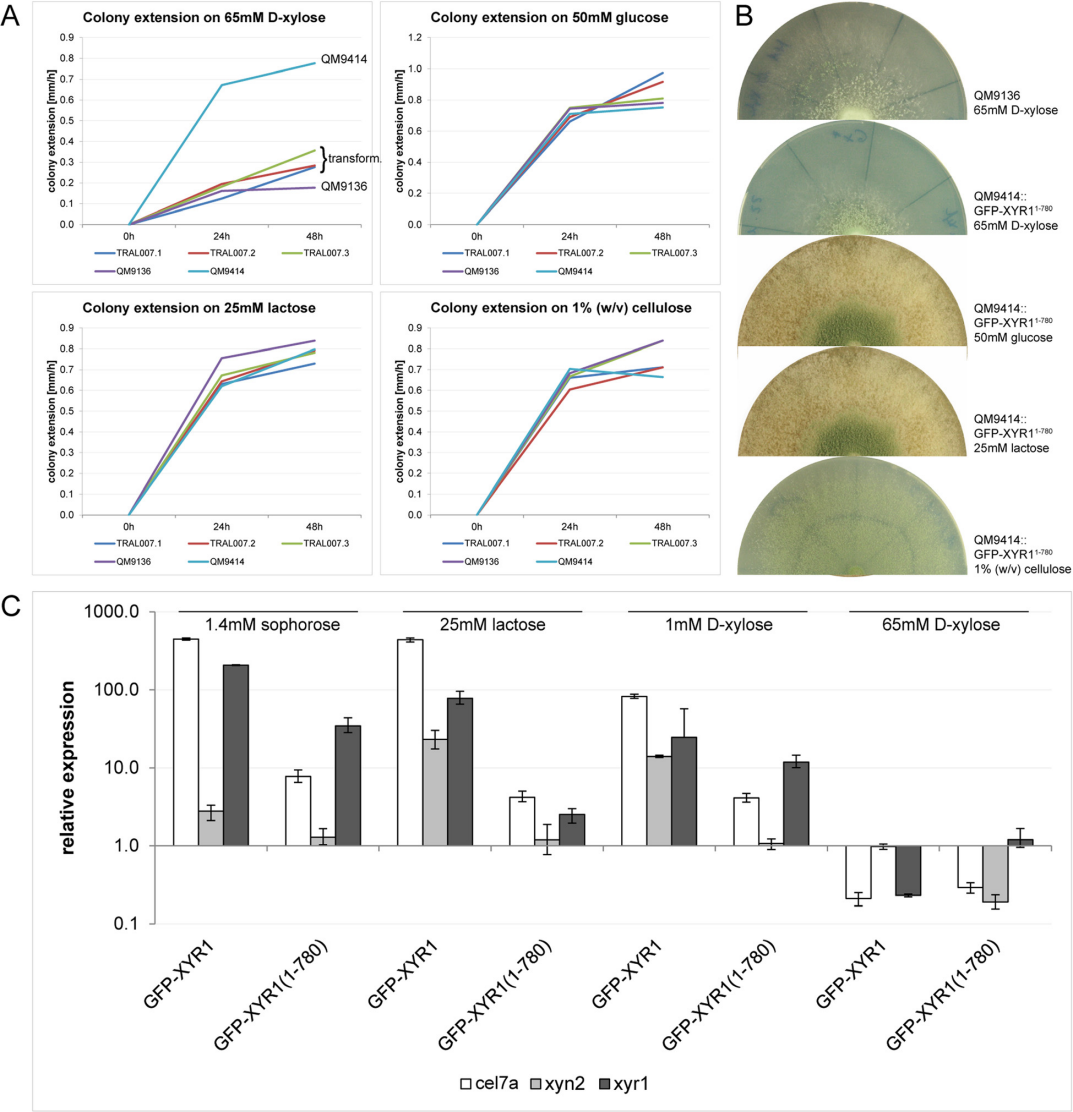
Gene replacement with the truncated GFP-XYR1^1–780^ reproduced the XYR1-loss-of-function phenotype in QM9414. **(A)** QM9414::GFP-XYR1^1–780^ transformants (TRAL007 clones 1 to 3) showed the same growth defect on 65 mM D-xylose as QM9136 and ∆*xyr1*, respectively, indicating loss of XYR1 function, which was further verified by the corresponding macroscopic colony phenotypes, shown in **(B)**. **(C)** Gene expression of cellulase *cel7a*, xylanase *xyn2* and *xyr1* were not significantly upregulated in GFP-XYR1^1–780^ expressing transformants (TRAL007), whereas the GFP-XYR1 transformants (TRAL002) clearly responded to the inductive stimulus provided by sophorose. Error bars show standard deviation (n = 6 from three experimental and two biological replicates involving two different TRAL007 clones).

The above data imply that the C-terminal 140 amino acids are essential for the function of XYR1. In order to test this directly, we replaced the wild type *xyr1* locus in *T. reesei* QM9414 with the corresponding *xyr1*^∆*A2294*^ allele containing the truncating mutation. The resulting transformants (TRAL007: QM9414∆t*ku70*, P*xyr1*::*gfp-xyr1*^∆*A2294*^::T*xyr1*) showed the same growth defect on D-xylose and macroscopic mutant phenotype as found in QM9136 (Figure 4A and B).

In addition, cellulase and xylanase gene expression was impaired in *T. reesei* expressing GFP-XYR1^∆A2294^ (Figure 4C). Expression of the gene encoding *cel7a* after 1 h induction with sophorose, for instance, was 60-times lower when the truncated XYR1 version was present. *Xyr1* expression, on the other hand, was only 6-times less in the mutant with truncated XYR1. We therefore conclude that presence of the genetically engineered, truncated XYR1^1–780^ is sufficient to reproduce the QM9136 mutant phenotype.

### Full-length XYR1 is transported into the nucleus in *T. reesei* QM9136

The loss of a conserved domain in a transcription factor can have three main consequences: (a) impaired stability and degradation; (b) lack of transport into the nucleus; and (c) lack of interaction with other factors responsible for enabling expression of the respective target proteins. Binding to DNA was not investigated because of the intactness of the Zn_2_Cys_6_ zinc finger of XYR1 in strain QM9136.

**Figure 5:**
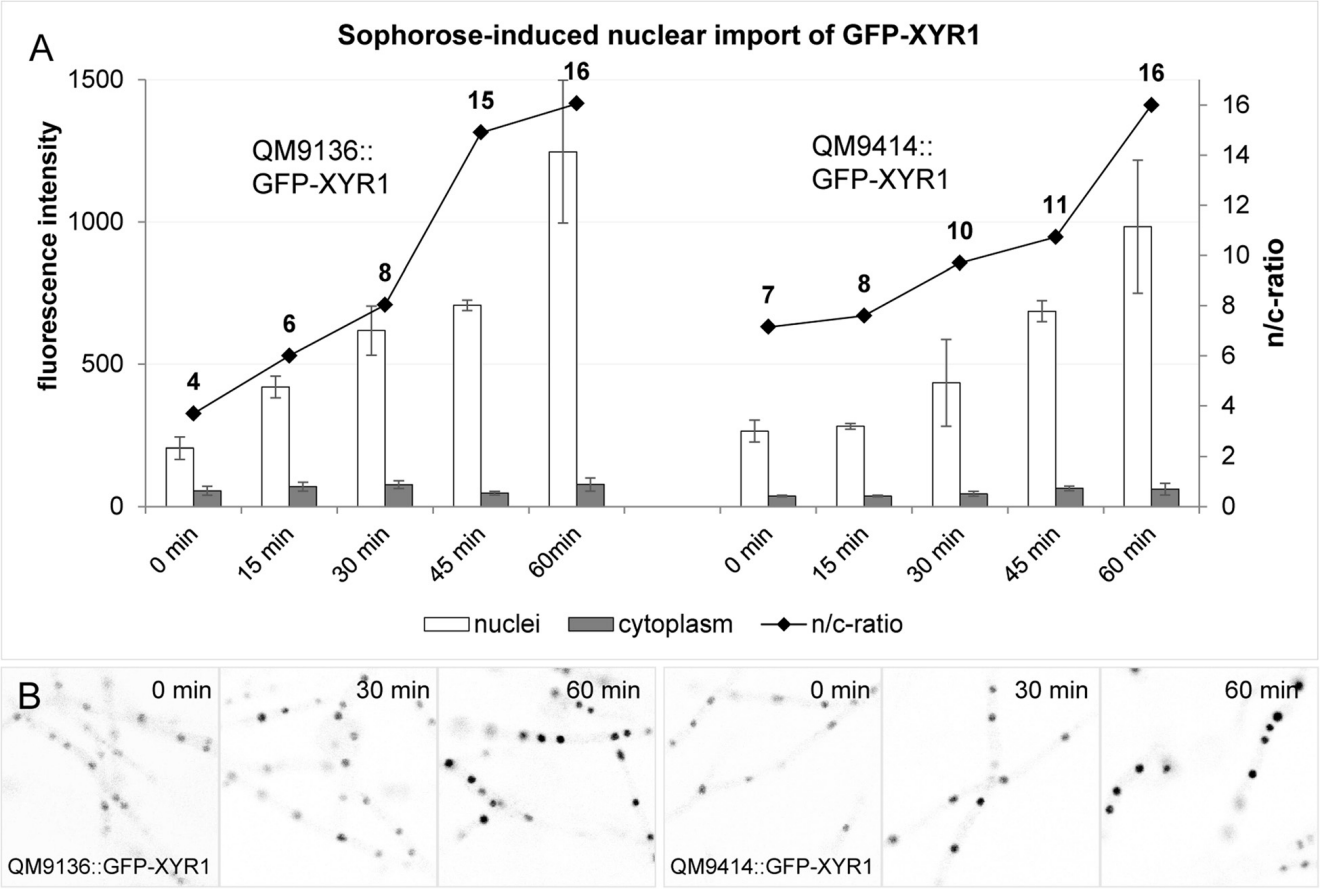
Nuclear import of GFP-XYR1 is indistinguishable in QM9136 and QM9414 transformants. **(A)** Sophorose-induced nuclear import of GFP-XYR1 in QM9136 and QM9414 backgrounds was indistinguishable. Relative fluorescence intensity represents the average GFP-XYR1 signal detected measured in 120 nuclei per sample. The nucleo-cytoplasmic ratio (n/c-ratio) provides a measure of how much brighter the average GFP-XYR1 signal is inside nuclei compared to the surrounding cytoplasm. Error bars represent standard deviation (n = 120). **(B)** Representative, inverted fluorescence images showing increasing GFP-XYR1 recruitment into nuclei of *T. reesei* germlings over a time course of 60 min after carbon source replacement with 1.4 mM sophorose.

In order to test (a) and (b), we employed N-terminal GFP-tagging of XYR1 (see above) and performed quantitative live-cell imaging analyses under cellulase inducing and non-inducing conditions. The necessity of an N-terminal GFP fusion, and the basic nucleo-cytoplasmic shuttling dynamics of XYR1 during cellulase and xylanase expression have previously been established by us [19]. As can be seen in Figure 5A and B, the dynamics of nuclear recruitment of GFP-XYR1 in response to the presence of the inducer sophorose, were indistinguishable in QM9136 and QM9414 transformants.

**Figure 6:**
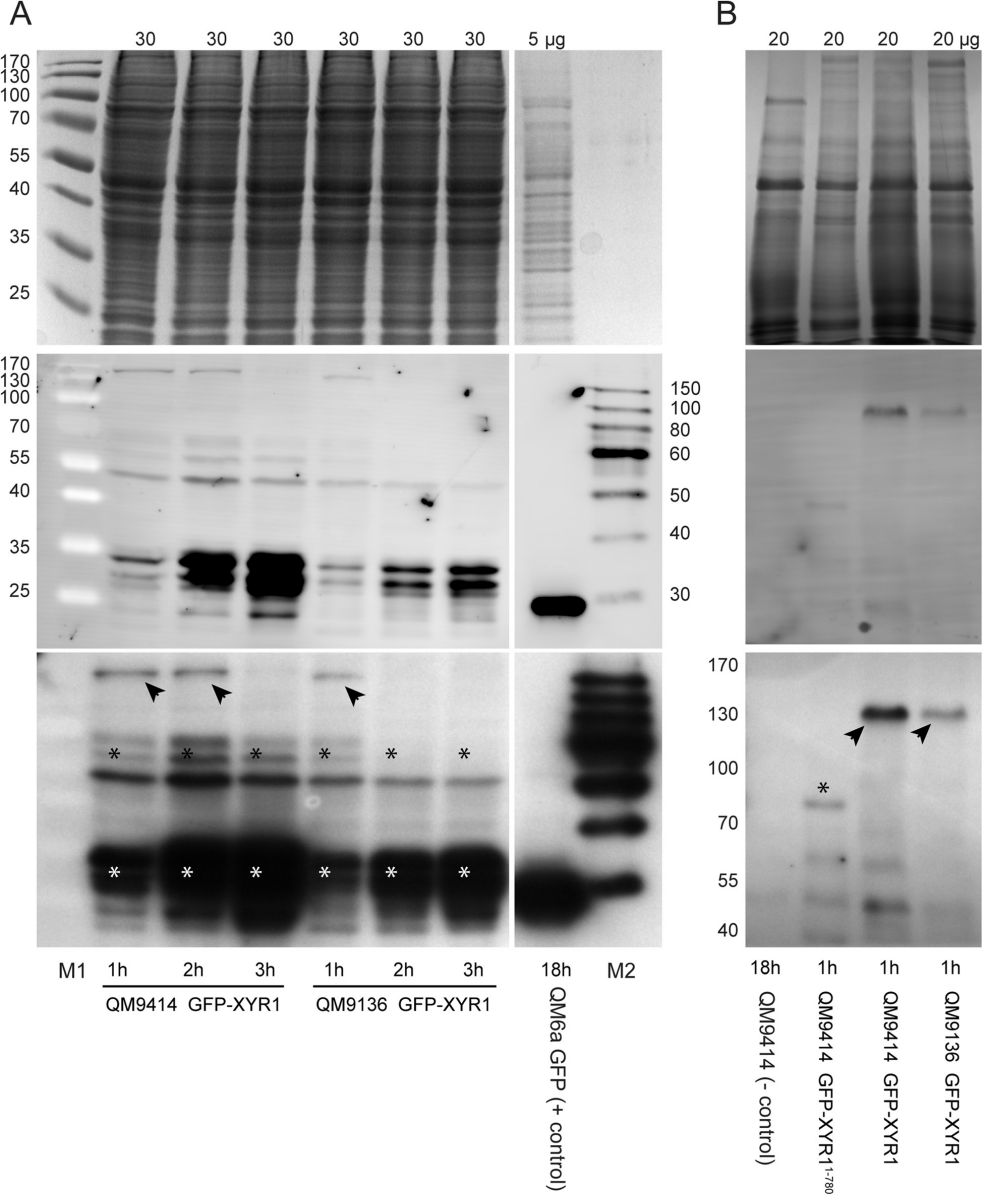
Western blot analysis of GFP-XYR1 and GFP-XYR1^1–780^ under cellulase inducing conditions. Top row: Coomassie stained loading control; middle row: chemifluorescent image of Western blot membrane; bottom row: x-ray image of Western blot membrane. **(A)** With time (1-3 h of sophorose induction), less of the 127 kDa full-length GFP-XYR1(arrowheads) but more of its various 30-70 kDa degradation products (asterisks) can be detected in protein extracts. This rapid turn-over of the transcription factor is conserved in QM9414 and QM9136 transformants. **(B)** In comparison to full-length GFP-XYR1 (arrowheads) expressed in QM9414 and QM9136 transformants, of the truncated GFP-XYR1^1–780^ construct, only ~80 kDa or smaller degradation products could be detected (asterisk). 5-30 μg refer to the total protein load per lane; numbers on the side denote molecular weight ranges; M1 is PageRuler pre-stained molecular weight marker; M2 is SuperSignal enhanced protein ladder; 18 h QM6a GFP is the positive control, and 18 h QM9414 the negative control for αGFP(B-2) antibody specificity.

Because reliable antibodies directed against XYR1 are currently not available, we furthermore took advantage of the GFP-tag to detect GFP-XYR1 by Western blot analysis, and confirmed that the high turn-over dynamics of the transcription factor previously observed in QM9414 [19], are conserved in the complemented QM9136 transformant, verifying functional rescue (Figure 6A). Summarizing, these data show that the cellular machinery required for nuclear import of XYR1 is generally intact in QM9136.

### The truncated XYR1^1–780^ protein is also transported into the nucleus

We consequently tested whether the mutated *xyr1*^∆*A2294*^ present in QM9136 would be expressed, and - if so – whether the resulting XYR1^1–780^ is transported into the nucleus. To this end, we again took advantage of GFP-labelling, and studied the intracellular localization dynamics of the GFP-XYR1^1–780^ construct with which we had replaced the native *xyr1* in *T. reesei* QM9414 (TRAL007).

**Figure 7:**
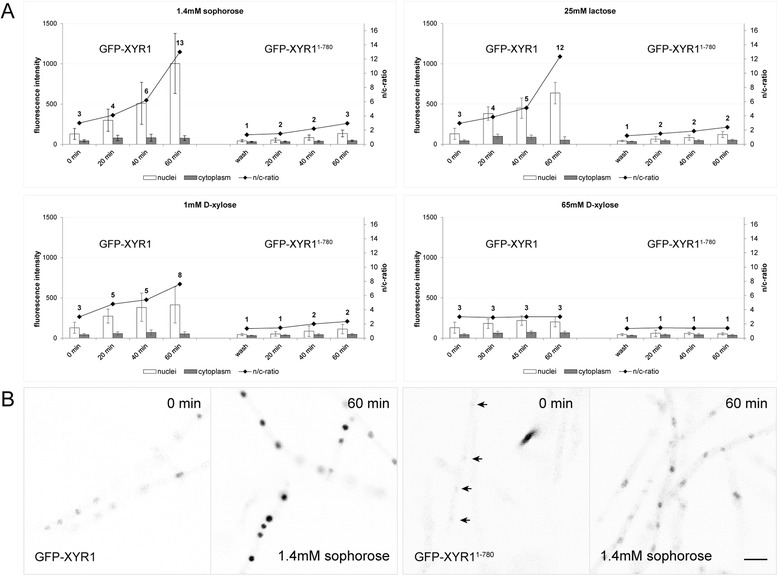
C-terminal truncation renders GFP-XYR1^1–780^ non-responsive to inductive signals. **(A)** Nuclear recruitment dynamics assessed by quantitative live-cell imaging. In contrast to the full-length GFP-XYR1 (TRAL002), whose concentration in nuclei rapidly increased by a factor of up to eight-fold, the truncated GFP-XYR1^1–780^ (TRAL007) was unable to significantly increase its presence in the nucleus in response to cellulase- and xylanase-inducing carbon sources (1.4 mM sophorose, 25 mM lactose and 1 mM D-xylose). Instead, GFP-XYR1^1–780^ consistently showed nuclear presence comparable to the basal presence of GFP-XYR1 under *xyr1*-non-inducing conditions (65 mM D-xylose). Error bars show standard deviation (n = 2 from two biological replicates involving two different TRAL007 clones). **(B)** Representative, inverted fluorescence images showing nuclear import of GFP-XYR1 and GFP-XYR1^1–780^ after one hour of sophorose induction. In contrast to the full-length GFP-XYR1 construct, the truncated version is barely visible inside nuclei before induction (arrows). Most importantly, upon addition of 1.4 mM sophorose, the nuclear signal of GFP-XYR1^1–780^ does increase, however, only to a fraction of the intensity (about 15%) of the GFP-XYR1 construct under identical conditions. Scale bar, 10 μm.

Nuclear import of truncated GFP-XYR1^1–780^ in response to an inductive signal was strongly decreased as compared to the full-length GFP-XYR1, nevertheless, still occurred at a low level. In comparison to the full-length construct which increased eight-fold within 60 minutes after replacement on sophorose, nuclear GFP-XYR1^1–780^ recruitment increased only three-fold, reaching just 13% of the average GFP-XYR1 concentration (Figure 7A and B). The inability of truncated XYR1 to adequately respond to an inducing signal was observed on all tested inductive carbon sources, and indirectly correlated with the efficacy of induction (*i.e.* 1.4 mM sophorose < 25 mM lactose < 1 mM D-xylose). Western blot analysis showed that in contrast to QM9414 and QM9136 transformants, in which full-length GFP-XYR1 (128 kDa) was readily detectable after 1 h of sophorose induction, only ≤ 80 kDa large degradation products of the truncated GFP-XYR1^1–780^ (actual size 112 kDa) could be detected (Figure 6B). This finding suggests that the truncated XYR1 protein undergoes proteasomal degradation much faster than the full length version, probably because it is quickly detected as non-functional.

### Basal XYR1 formation is unaffected by the *xyr1*^∆*A2294*^ mutation

Despite the strong reduction of the degree and rate of nuclear uptake in the strain harbouring the *xyr1*^∆*A2294*^ allele, the cytosolic level of the truncated XYR1 remained low but comparable to that in the parental strain (Figure 7A). In order to rule out that this would be an artefact caused by autofluorescence of *T. reesei*, we repeated these experiments under microscope settings optimized to completely eliminate cellular background auto-fluorescence detection (see Methods). Under these stringent detection settings, any fluorescence signal must emanate from GFP only. As shown in Additional file 5: Figure S4, the fluorescence intensity of cytosolic GFP-XYR1^1–780^ was the same under XYR1 inducing and non-inducing conditions (1 mM and 65 mM D-xylose, respectively), and was also similar to that measured in *T. reesei* QM9414 expressing the fully functional GFP-XYR1. Interestingly, in this experiment we detected GFP-XYR1^1–780^ in small cellular spots under inducing conditions, which we hypothesize might be clusters of proteasomal degradation leaving the more stable N-terminal GFP behind. This is in line with our previously reported finding, that the GFP moiety of the fusion protein remains longer inside nuclei than the much more rapidly degraded XYR1 part [19]. Summarizing, we conclude that basal expression of *xyr1* is not affected by the C-terminal truncation of *XYR1*^*1–780*^ present in *T. reesei* QM9136.

### Impaired nuclear recruitment of XYR1^1–780^ occurs throughout the whole fungal colony

The results documented in Figure 7 illustrate that the truncated GFP-XYR1^1–780^ is indeed transported into the nucleus at the same rate as the full length GFP-XYR1, albeit the ratio of nuclear-to-cytoplasmic accumulation was reduced by > 75%. As these experiments were conducted with germinating liquid cultures, we wondered whether this reduction in GFP-XYR1^1–780^ biosynthesis and nuclear import would also occur throughout the mature fungal colony – *i.e.* in the colony periphery comprised of fast growing hyphae, the colony subperiphery which displays prolific hyphal branching, and the central area of the colony which is additionally characterized by aerial hyphae formation and prolific sporulation. We have recently shown that the central region of the colony was the one with the highest nuclear concentration of XYR1 [19].

**Figure 8:**
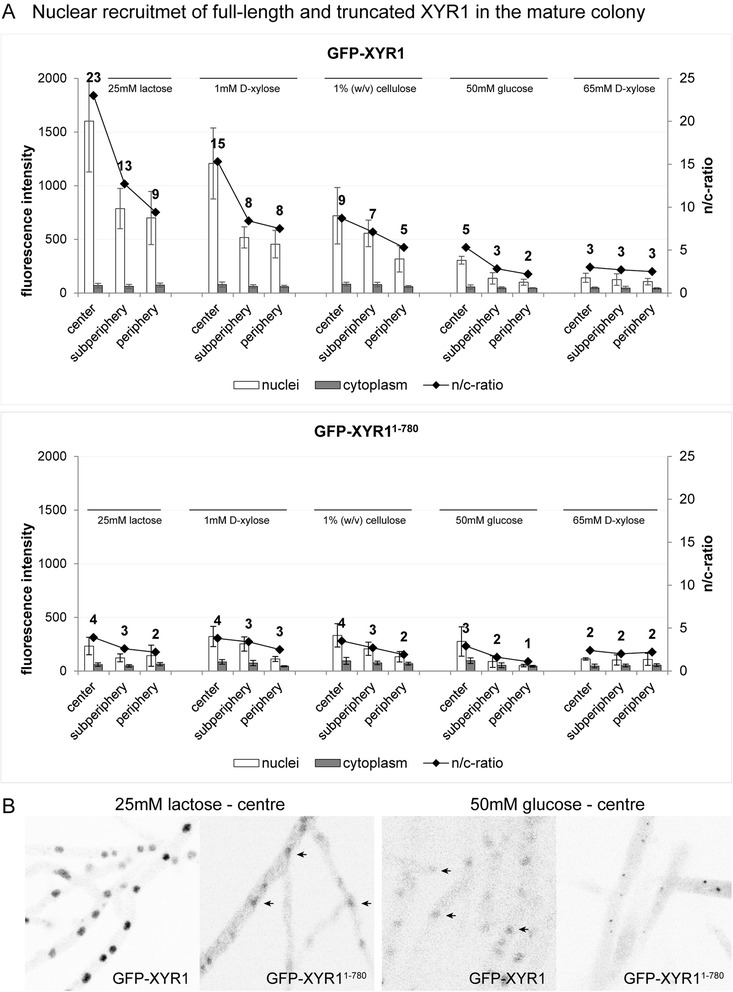
Quantification of nuclear recruitment of GFP-XYR1 and GFP-XYR1^1–780^ in the three main functional zones of the mature colony. All strains were grown for 48 hours on MA medium agar plates supplemented with the indicated carbon source. **(A)** Full-length GFP-XYR1 responds to different *xyr1*-inducing signals with nuclear recruitment, most prominently in the central area of the colony, and dependent on the inductive strength of the provided carbon source (25 mM lactose > 1 mM D-xylose > 1% (w/v) cellulose). Under non-inducing/repressing conditions (50 mM D-glucose or 65 mM D-xylose) only weak baseline nuclear recruitment can be detected. In contrast, the truncated GFP-XYR1^1–780^ does not significantly respond to cellulase induction, and hence can only be detected at baseline levels independent of the provided carbon source. **(B)** Representative, inverted fluorescence images of the tested conditions, showing that GFP-XYR1^1–780^ does respond to 25 mM lactose with weak but detectable nuclear import, comparable to the baseline presence of GFP-XYR1 under repressing conditions (barely visible nuclei are indicated with arrows). On 50 mM D-glucose GFP-XYR1^1–780^ is visible in small spots, suggesting degradation of the non-functional XYR1 construct in the proteasome.

Strains expressing the full-length GFP-XYR1 behaved as expected: strong nuclear recruitment of XYR1 with a clear intensity peak in the colony centre on *xyr1-*inducing carbon sources, and essentially the same profile but with about 10-times weaker peak intensities on *xyr1-*non-inducing carbon sources (Figure 8A and B). The strain expressing the truncated GFP-XYR1^1–780^ on the other hand, showed low-intensity profiles independent of the used carbon source. Notably, the overall amounts of GFP-XYR1^1–780^ on any carbon source tested were comparable to those of GFP-XYR1 under non-inducing conditions, suggesting that the induction-independent baseline production of GFP-XYR1^1–780^ functioned normally, however, was unresponsive to inducing signals. This was in line with data collected in shuttling experiments using conidial germlings in liquid culture. Taken together these results suggest, that C-terminal truncation of XYR1 prevents any XYR1-dependent cellular response, independent of the used carbon source, the developmental age of the fungus, *i.e.* germling vs. mature colony, or the functionally stratified colony region.

## Discussion

Historically, the cloning of genes was based on the use of complementation of mutants, which favored those organisms which either contained plasmids or autonomously replicating elements (for easy recovering of the target gene) or which could efficiently be crossed in the laboratory. Although a number of substitute strategies had been developed (cf. [20]), this nevertheless strongly limited the progress in organisms that exhibited neither of these possibilities, such as the so-called imperfect fungi. The advances in sequencing technologies and bioinformatics have now made it possible to sequence even full eukaryotic genomes quickly, and to use this strategy to identify mutations responsible for a particular phenotype [21-26]. However, since mutant organisms often contain several mutations (cf. [5]), most researchers either mapped the target mutation or backcrossed their mutant against the wild type, and sequenced more than one mutant genome simultaneously.

Since the *T. reesei* mutants were maintained asexually, neither of these two strategies was possible for the present study. Hence, we were fortunate that the cellulase-negative strain QM9136 only exhibited eight mutations, of which several could be characterized as unlikely candidates for this phenotype. In accordance with the fact that *T. reesei* QM9136 arose from X-ray treatment (linear accelerator), which predominantly gives rise to elimination of nucleotides, we found a higher number of indels than SNVs. Vitikainen *et al.* [6] made the same observation for the *T. reesei* strains QM9414 and QM9123, which arose also by X-ray treatment [3,9], whereas the *T. reesei* NG14 and RUT C30, which were isolated by UV light-treatment [27], exhibited a higher number of SNVs [5,6]. We found no bias in the occurrence of the mutations in the genome, as all eight occurred on different scaffolds. Yet it was intriguing to note that all but one were located at the ends of the scaffolds, and five of them occurred at scaffolds of < 1 Mbp size. Since the size of the scaffolds is determined by the presence of long and non-alignable nucleotide repeat regions, it is possible that the proximity to regions of genomic instability facilitates the susceptibility to mutation by X-rays.

Using gene complementation, we showed that a truncation in XYR1, caused by a single nucleotide deletion, is very likely the exclusive reason for the cellulase-negative phenotype of *T. reesei* QM9136. XYR1 is the main actor among at least four positive transcriptional activators (XYR1, ACE2, ACE3 and the HAP2/3/5 complex), because its knock-out not only eliminates cellulases but also xylanase and ß-mannanase formation (for review see [28-30]). This finding is also nicely reflected in the phenotype of QM9136, which fails to grow on 65 mM D-xylose (on agar plates and in liquid culture), has strongly reduced or no growth on lactose and cellulose, respectively, (both only in liquid culture) [31], and is deficient in the formation of cellulases, xylanases and mannanases [10].

While the identification of a gene (*xyr1*) that is already known to be crucially involved in cellulase and hemicellulase formation, as the cause for the inability of cellulase formation of *T. reesei* QM9136, may be considered trivial, it nevertheless sheds new light into its molecular function: XYR1 belongs to the fungal-specific Zn_2_Cys_6_-binuclear zinc cluster transcription factor family [32,33]. The highly conserved domain structure of this protein family contains – besides the zinc cluster domain recognizing the nucleotide binding motif – a fungal transcription factor regulating middle homology domain (FTFRMH; [34]), and short acidic activation domains within the distal portion of the C-terminus. With regard to XYR1, these domains have not yet been clearly located [35]. In this study, we have delimited these domains and consequently presented evidence that the XYR1 truncation in *T. reesei* QM9136 exclusively removed the C-terminal acidic activation domain. This implies that this domain is essential for the activation of cellulase, xylanase and ß-mannanase gene expression. In fact, two mutations in the XYR1 ortholog XlnR from *A. niger* (L823S and Y864D) are also located in the acidic C-terminal region and also resulted in an impairment of xylanase gene expression [36,37]. We therefore conclude that this region is essential for binding the proteins or other compounds that mediate induction of cellulase and hemicellulase formation in *T. reesei*. Interestingly, the above mutations lie within the four short helices that form the C-terminal coiled-coil. In the *Arabidopsis thaliana* TCP8 Zn_2_Cys_6_-type transcription factor this coiled-coil acts both as a transactivation and self-assembly domain [38]. Since the nucleotide motif that binds XYR1 has in most cases been described to only function *in vivo* when present as a direct or indirect repeat [39-41], we hypothesize that XYR1 has to form a dimer. A mutation in or absence of this coiled-coil domain therefore may impair binding of XYR1, and thus cellulase and hemicellulase gene expression.

Interestingly, two mutations have been reported in the N-terminal part of the acidic activation domain that result in the opposite, *i.e.* constitutive expression of xylanases in *A. niger* and *T. reesei* and elevated basal expression of cellulases in *T. reesei* only. These are V756F in XlnR (corresponding to V801 in XYR1) and A804V (based on our analysis; not A824V as stated by the authors) in XYR1 [35,42]. Since truncation of XYR1 in QM9136 starts before both positions, this implies that the above mutations are only functional when the whole acidic activation domain is present. One model that would explain these findings is that V801 and A804 are binding a repressor of xylanase induction, which is antagonized by binding of the proposed activator. According to Derntl *et al.* [35], induction of cellulase gene expression is not altered, but their basal expression level is increased. In *T. reesei*, the cellulase genes *cel7a* and *cel6a* as well as the xylanase genes *xyn1* and *xyn2* are regulated by carbon catabolite repression mainly at the basal transcription level [43,44]. It would thus be tempting to speculate that V801 and A804 are necessary to bind the respective catabolite repression regulator CRE1 [45,46], whose DNA-binding sites in the above genes are in close vicinity to those for XYR1 [43,44]. However, the strain of Derntl *et al.* [35], in which this increase of the basal level of cellulase transcription has initially been detected, is a derivate of *T. reesei* RUT C30 which bears a nonfunctional *cre1* copy [46]. The nature of the repressor binding at V801 and A804 would thus need to be identified first.

We have previously shown that *xyr1* is transcribed at a low level constitutively, but its expression is enhanced in the presence of one of the known cellulase or xylanase inducers [19,47]. This constitutive low concentration of XYR1 is already transported into the nucleus, and we could show here that this also occurs in the QM9136 mutant. We therefore conclude that the C-terminal acidic domain does not contain a trigger for nuclear transport. These findings are generally in accordance with those of Hasper *et al.* [42] who found that truncation of a C-terminal region of *A. niger* XlnR including the coiled-coil of the fungal transcription factor activation domain (FTFAD) leads to lower but still clearly detectable nuclear localization. However, in contrast to the truncation in XlnR, the truncated XYR1 in QM9136 still bears an intact FTFAD-terminal coiled-coil region. Our data therefore suggest that the putative regulatory C-terminus of XYR1 is not *per se* required for nuclear import of the transcription factor, but rather appears to be involved in its induction-dependent, positive auto-regulatory feedback mechanism. This explains why the fluorescent intensity of the truncated XYR1 protein stays generally low because the protein cannot be upregulated by its own production and achieve sufficient nuclear accumulation to trigger cellulase and hemicellulase gene expression.

## Conclusions

Our study demonstrates how high-throughput genomic sequencing combined with reverse genetics can provide novel functional insights into organisms that are otherwise only poorly susceptible to mutational analyses. The mutation in *xyr1* in *T. reesei* QM9136 also allowed new insights into the function of this central cellulase regulator. The ongoing investigation of yet two more cellulase-negative strains of *T. reesei* (*i.e.* QM9978 and QM9979; [48]) – which bear a functional *xyr1* copy (C.P. Kubicek, B. Seiboth, A. Margeot and S.E. Baker, unpublished data) – by a similar approach is expected to further elucidate cellulase and hemicellulase regulation in this important filamentous fungus.

## Methods

### Strains and growth conditions

The *Trichoderma reesei* strains QM6a (ATCC13631), the ex-type isolate [49] QM9136 (ATCC 26920), a mutant defective in cellulase production [9,10], QM9414 (ATCC 26921), an early cellulase producing mutant derived from isolate QM6a, and its ∆*xyr1* knock-out strain [12], were used throughout this work. *T. reesei* transformant strains expressing green fluorescent protein (GFP)-labeled XYR1 fusion proteins were generated in a QM9414 background strain which had the *ku70* gene (responsible for non-homologous recombination by end-joining DNA) removed [50]. The used parental strain (QM9414∆*tku70*) was constructed similar to the ∆*ku70* strains as described previously [50], with the only difference that the selection marker for the targeted *ku70* gene knock-out was the *ptrA* gene [51].

Strain propagation, transformant selection and purification were performed on potato dextrose agar (PDA) supplemented with suitable selection agent when required. For submerged cultures, strains were grown in Mandels-Andreotti (MA) medium [52], using D-glucose, D-xylose, glycerol, lactose, sophorose or cellulose as sole carbon sources at final concentrations for up to 1% (w/v) as indicated. All strains are maintained as 50% (v/v) glycerol stocks at −80°C. Table 2 lists all strains used and generated in this study.

**Table 2:** ***T. reesei*** **strains used and produced in this study**

**Strain**	**Strain number**	**Parental strain**	**Genotype**	**Reference**
QM9414	ATCC26921	QM6a	-	[52]
QM9414 Δ*tku70*	-	QM9414	Δ*ku70*::*ptrA*	this study
GFP	-	QM6a	P*tef1::gfp*	[19]
Δ*xyr1*	-	QM9414	Δ*xyr1::hph*	[12]
GFP-XYR1	TRAL002	QM9414 Δt*ku70*	Δ*ku70*::*ptrA*;Δ*xyr1*::*hph*; P*xyr1*::*gfp*-*xyr1*::T*xyr1*	[19]
GFP-XYR1^1–780^	TRAL007	QM9414 Δt*ku70*	Δ*ku70*::*ptrA*;Δ*xyr1*::*hph*; P*xyr1*::*gfp*-*xyr1* ^*∆A2294*^::T*xyr1*	this study
GFP-XYR1	TRAL008	QM9136	Δ*xyr1* ^*∆A2294*^::*hph*; P*xyr1*::*gfp*-*xyr1*::T*xyr1*	this study

Minimal medium [53] supplemented with 1–2% (w/v) sugars (D-glucose, cellobiose, lactose or cellulose) were used to determine growth phenotypes on agar plates. Plates were centrally inoculated with small agar blocks carrying mycelium, and incubate for 3–5 days at 30°C.

*Escherichia coli* strains JM109 (Promega, Madison, Wisconsin), One Shot®Top10 (#C4040-10, Life Technologies-Invitrogen, Austria) or Stellar® (#636763,Takara Bio Europe/Clontech, Saint-Germain-en-Laye, France) were used for plasmid construction and amplification using standard molecular cloning techniques [54].

### Illumina genome sequencing of *T. reesei* QM9136

Chromosomal DNA from *T. reesei* QM9136 was prepared as described previously [5]. Fragment libraries were prepared according to the TruSeq® DNA Sample Preparation Guide from Illumina (www.illumina.com). These libraries were then loaded onto the cluster generation station for single molecule bridge amplification using the Standard Cluster Generation kit from Illumina. The slide with amplified clusters was then subjected to sequencing on the Illumina Genome Analyser I (GAI) for single reads using the 36 cycle Sequencing Kit version 1 from Illumina.

The whole genome sequence of *T. reesei* QM9136 was deposited at the Sequence Read Archive (SRA; http://www.ncbi.nlm.nih.gov/sra) under the accession number SRX059777.

### Sequence alignment and analyses

Illumina short reads from QM9136 were mapped onto the *T. reesei* genome (http://genome.jgi-psf.org/Trire2/), using the Maq 0.6.6 software solution [55]. Mapping was done with two maximum mismatches. InDels and SNVs were also identified using Maq 0.6.6. Homozygous mutations were selected and filtered on genomic context (complexity = 1, uniqueness > 15.8, GC percentage between 0.31 and 0.74). Each mutation was manually checked using the Integrative Genome Viewer and compared to sequence of the RUT lineage (Figure 1) to remove the false mutation coming from initial QM6a error sequencing. Large structural variations were searched by using the BreakDancer software [56] and filtered on the reads number covering the genomic variations.

We evaluated the location of SNVs and deletions according to gene annotations using the “filtered models” from the JGI website. From this annotation we calculated the position of intron, promoter (using an 800 bp upstream region) and terminator (using a 200 bp downstream region).

### Generation of gene replacement cassettes

To express GFP-labeled versions of full-length and truncated XYR1 transcription factor from its native locus, gene replacements cassettes were constructed that exchanged the native *xyr1* open reading frame with full length or genetically truncated copies of each *gfp*-fusion gene and the hygromycin resistance expression cassette marker gene ([57]; Additional file 6: Figure S5). C-terminal truncation of XYR1 was achieved by reproducing the deletion of A2294 (∆A2294) identified in the QM9136 genome through side-directed mutagenesis in the *xyr1* locus of the replacement cassette, leading to the expression of a GFP-XYR1^1–780^ construct in the resulting transformants. Homologous recombination was facilitated via the native ~1 kb 5′ and 1 kb 3′ flanking regions. Assembly of the individually generated amplicons from *T. reesei* genomic DNA was performed using InFusion® recombinational PCR cloning (HD #639649, Takara Bio Europe/Clontech, Saint-Germain-en-Laye, France). In-frame cloning of all four gene replacement cassettes was verified by nucleotide sequencing of PCR-amplicons generated from extracted, genomic DNA of the respective transformants.

### *T. reesei* transformation and genotyping by PCR and sequencing

Gene replacements cassettes were amplified from the respective plasmids by PCR and transformed into *T. reesei* QM9414*∆tku70* as linear DNA fragments using electroporation as described previously [58]. Transformant strains were selected on PDA hygromycin medium (100 μg/ml hygromycin final concentration), and homokaryons were obtained by repeated rounds of vegetative spore propagation on selective medium. Individual isolates were genotyped for integration of the replacement cassette at the target gene locus by PCR as described in detail elsewhere [59]. Briefly, primer pairs, binding inside and outside of the replacement cassette and native ORF, respectively, were used to probe for: (1) the integration of the replacement cassette at the target gene locus, (2) the presence of the *hph*-resistance marker cassette at this locus, (3) the presence of GFP at this locus, and (4) the absence of the target ORF from the whole genome. When reactions were performed as multiplex PCRs, an additional pair of oligonucleotides binding within the actin locus (Trire2:44504) was used as an internal positive control for each reaction. Selected amplicons resulting from PCR genotyping were furthermore subjected to DNA sequencing, notably for verification of the *xyr1*^∆*A2294*^ point deletion in TRAL007 transformants. Table 3 lists all oligonucleotides used for PCR genotyping and DNA sequencing in this study.

**Table 3:** **Oligonucleotides used in this study for PCR genotyping and DNA sequencing**

**Primer name**	**5′-3′ sequence**
*PCR genotyping*	
Pxyr1-ver-F	AAGGATGCCGACTTAACGAAC
Txyr1-ver-R	AGTCGCTCATGATCCTACCAG
Xyr1-ver-R	CCTGGCAGCAATAAGAGAGC
Xyr1-ver-F	CCTTGCGGATAAGTGGGATC
5Pxyr1-ver-F	CCAGCTGCCACTCTCATG
GFP-ver-R	AAGCACTGCACGCCGTA
GFP-ver-F	TACGGCGTGCAGTGCTTC
Pgpdh-ver-F	TGCTAAGGTACCTAGGGAGGGA
hph-ver-R	CAAGCACTTCCGGAATCG
act-mp-F	ACTTTCGGCCGCATTCTG
act-mp-R	AGCCAGGATCTTCATCAGGTAG
*DNA sequencing*	
Pxyr1-seq-F	GACAGCAGCAGTAGTCAGGT
Txyr1-seq-F	TTGCTGAAAGTGAAGAGGG
xyr1-seq-F	CCGTCTCCCAAGACTAGC
5′xyr1-seq-F	CACATTGAGGGCTCTGTC
3′xyr1-seq-F	AAGCATCTGCCATTGTCC
Xyr1∆A2294-seq-F	GCCACGATGCAGGGAA
3′GFP-seq-F	CCGACAACCACTACCTGA
5′GFP-seq-F	GCCACAAGTTCAGCGTG
Pgpdh-seq-F	CATGCAGTTGCTAAGGTACC
Tgpdh-seq-R	GACTGTGAGCATGTGATGGC

### Real-time monitoring of XYR1 nuclear transport by quantitative live-cell imaging

In order to monitor rapid changes in the subcellular localization of GFP-tagged XYR1, carbon source replacement experiments were performed using submerged germling cultures. For this, conidia from one week-old carbon source-free MA plate cultures were harvested in sterile water and cell concentration determined using a Thoma cell counting chamber. One hundred million cells were used to inoculate 100 ml MA pre-culture medium (liquid MA medium with peptone to aid germination) in 500 ml shake flasks, supplemented with either 50 mM D-glucose (cellulase repressing condition) or 1% w/v carboxymethyl-cellulose (cellulase inducing condition) as sole carbon source, and incubated at 28°C and 200 rpm overnight in the dark. The next morning biomass from 20 ml pre-culture aliquots were washed twice with sterile tap water and transferred into 20 ml carbon source-free MA replacement medium (liquid MA medium w/o peptone) in 100 ml shake flasks. If desired, germlings were starved for up to one hour under identical incubation conditions in order to drain internal carbon storage. At time point t = 0 min a new carbon source for cellulase induction (1.5 mM sophorose, 1 mM D-xylose, 25 mM lactose or 1% w/v cellulose) was added and incubation continued. Cell samples for microscopical analysis were collected from overnight pre-cultures before and after carbon source replacement at desired time points. Carbon source-free MA cultures were used as controls for de-repressing conditions.

Expression and subcellular localization of GFP-labeled fusion proteins was quantified using scanning confocal microscopy and image analysis as described in detail recently [19]. Briefly, average nuclear and cytoplasmic fluorescence intensities were calculated from at least 120 individual measurements per experimental condition (using fixed-size, circular measuring tools placed onto optically sectioned nuclei or into the adjacent cytoplasm) and graphically evaluated. Standard deviation bars in the respective graphs represent the considerable biological variation of transcription factor recruitment to the individual, mitotically and transcriptionally non-synchronized nuclei within the population, rather than a true statistical error.

Live-cell imaging was performed using a Nikon C1 confocal laser scanning unit mounted on a Nikon Eclipse TE2000-E inverted microscope base (Nikon GmbH, Vienna, Austria). GFP-labelled proteins were excited with the 488 nm laser line of an argon ion laser, and the emitted fluorescence light separated by a Nikon MHX40500b/C100332 filter cube was detected with a photo-multiplier tube within the range of 500–530 nm. A Nikon Plan Apo VC 60×/1.2 water immersion objective lens was used, and laser intensity and dwell time during image acquisition were kept to a minimum to reduce photobleaching and phototoxic effects. Brightfield images were captured simultaneously with a Nikon C1-TD transmitted light detector mounted behind the condenser turret. Images were recorded with a maximum resolution of 1024×1024 pixels and saved as TIFF. Apart from display range adjustments, images were not subjected to further manipulation. Intensity measurements were performed with the MacBiophotonics ImageJ work package available at (www.macbiophotonics.ca/software.htm).

### Gene expression analysis

RNA extraction was performed according to [60], and if necessary purified using the RNeasy MinElute Cleanup Kit (Qiagen, Hilden, Germany). RNA quality and quantity were determined using a Nanodrop spectrophotometer (Thermo Scientific, Vienna, Austria). Transcript quantification was performed by RT-qPCR. To this end, DNase I-treated (Fermentas, #EN0521) RNA (3 μg) was reverse-transcribed with the RevertAid First Strand cDNA Kit (Thermo Scientific, #K1632) according to the manufacturer’s protocol with a 1:1 combination of the provided oligo-dT and random hexamer primers.

All RT-qPCR experiments were performed on an Eppendorf realplex^2^ Mastercycler (Eppendorf, Hamburg, Germany). Each sample was prepared as 25 μl reaction using the iQ SYBR Green Supermix (Bio-Rad, #170-8882) with a final primer concentration of 100 nM forward and reverse primer each. All assays were carried out as triplicates in a 96-well plate format covered with optical tape, including no-template controls. Measurements with the housekeeping gene translation elongation factor 1 (*tef1*) were performed for reference calculation and data normalization. Determination of the PCR efficiency was performed using triplicate reactions from a dilution series of cDNA (1; 0.1; 0.01; 0.001). RT-qPCR primers, amplification efficiency and R-square values are given in Table 4. Amplification efficiency of each sample mRNA was then calculated from the given slopes in the iQ5 Optical system Software v2.0 and fold-changes in gene expression were calculated using REST Software [61]. All samples were analysed in at least two independent biological experiments with three RT-qPCR replicates in each run.

**Table 4:** **RT-qPCR primers used in this study**

**Gene**	**Primer name**	**5′-3′ Sequence**	**R** ^**2**^	**Efficiency**
*cel7a*	qPCR-cel7a-F	CCGAGCTTGGTAGTTACTCTG	0.990	0.98
	qPCR-cel7a-R	GGTAGCCTTCTTGAACTGAGT		
*xyn2*	qPCR-xyn2-F	CAACCAGCCGTCCATCATCG	0.993	0.97
	qPCR-xyn2-R	ATCGTCCCGAGCGTCAGG		
*xyr1*	qPCR-xyr1-F	CCATCAACCTTCTAGACGAC	0.987	0.99
	qPCR-xyr1-R	AACCCTGCAGGAGATAGAC		
*cre1*	qPCR-cre1-F	GTCTGAGAAACCTGTCCCTG	0.996	0.91
	qPCR-cre1-R	GGCTAATGATGTCGGTAAGTG		
*tef1*	qPCR-tef1-F	CCACATTGCCTGCAAGTTCGC	0.995	0.95
	qPCR-tef1-R	GTCGGTGAAAGCCTCAACGCA		

### Protein extraction, SDS-PAGE and western blot analysis

Overnight liquid pre-cultures (100 ml MA medium, 1% w/v glucose) were prepared as described above for shuttling experiments. Samples for positive (QM6a; P*tef1*::*gfp*) and negative (QM9414 ∆*tku70*) controls were directly drawn from these pre-cultures after 18 h of incubation. Biosynthesis of GFP-XYR1 and GFP-XYR1^1–780^, respectively, was induced by transferring fungal biomass from 50 ml pre-culture into 50 ml fresh MA medium containing 1.4 mM sophorose. These induced cultures were harvested after 1, 2 and/or 3 hours of additional incubation. For each sample, fungal biomass from 50 ml liquid pre- or induced culture was harvested on a glass microfiber filter (Whatmann, cat.no. 1822–047) using vacuum-driven filtration, and, after washing twice with sterile tap water, immediately shock frozen in liquid nitrogen. Subsequently, the biomass was ground to a fine powder in liquid nitrogen, and approximately 100 mg of it were added to a 2 ml Eppendorf tube already containing 1 ml of protein extraction buffer (10 ml PBS containing 5 mM EDTA and 5 mM PMSF plus one cOmplete ULTRA protease inhibitor cocktail tablet (Roche, cat.no. 05 892 791 001), pH 7.4), and 1 g of small (0.25 mm diameter) and four large (3 mm diameter) glass beads to aid cell destruction. The mix was subjected to three one-minute rounds of homogenization at 30 Hz with one-minute cooling intervals at −20°C. Cell debris and aqueous phase were separated by centrifugation with 17.000 × g for 5 min and at 4°C. The clear supernatant containing all soluble proteins was transferred into fresh, pre-cooled Eppendorf tubes and stored at −20°C until further use. Total protein concentration was determined against BSA using Bradford reagent (BioRad, cat.no. 500–0006) according to manufacturer's recommendations. Typically, protein yields between 1–3 mg/ml were achieved.

Depending on the strain and protein of interest, *e.g.* for cytosolic GFP expressed under control of the constitutive P_*tef1*_ promoter in *T. reesei* QM6a much less total protein was required to yield a high detection signal on the Western blot, 5–30 μg of the crude total protein extract per lane were separated by SDS-PAGE as outlined in detail elsewhere [62]. Generally, two identical 12-14% SDS-PAGE gels were prepared, one for colloidal Coomassie staining, and the second for semi-dry electro blotting of the separated proteins onto ImmobileFL PVDF membrane (Millipore, cat.no. IPFL00010). Subsequent blocking was achieved by incubation in PBS-T (PBS, 0.3% Tween 20) supplemented with 2% w/v milk powder (Roth, cat.no. T145.1) for 1 hour at room temperature. For the specific labelling of GFP and GFP-fusion proteins, respectively, the monoclonal mouse anti-GFP-HRP antibody α-GFP(B-2) (Santa Cruz, cat.no. sc-9996) was used, diluted 1:1000 in PBS-T containing 0.5% w/v milk powder, and incubated on the membrane for 2 hours at room temperature, followed by four washing steps with PBS-T.

Detection of the labelled proteins was performed with the Pierce ECL2 kit (Thermo Scientific, cat.no. 80197) according to manufacturer's recommendations. Chemifluorescent signals were recorded on a Typhoon FLA700 imager (GE Healthcare), and chemiluminescent signals were visualized by x-ray film (Amersham Hyperfilm ECL, GE Healthcare, cat.no. 28-9068-35) exposure. For protein band size estimation, two molecular weight markers were used: PageRuler Pre-stained Protein Ladder 10 – 170 kDa (Thermo Scientific, cat.no. 26616), and SuperSignal Enhanced Protein Ladder 20 – 150 kDa (Thermo Scientific, cat.no. 84786), with only the latter one being applicable for ECL detection on x-ray film.

### Bioinformatic *in silico* analyses of XYR1

Functional domains were analysed by Pfam domain search [15]. Coiled-coil regions were identified using COILS [16], and Protparam [18]) was used for the determination of general physicochemical properties of XYR1. The 3D-functional domain structure of XYR1 was determined by the RaptorX protein structure prediction software (raptorx.uchicago.edu; [17]).

## Additional files

Additional file 1: Figure S1. Phenotypic comparison of QM6a and QM9136. Growth of *T. reesei* QM6a (parental strain) and QM9136 on solid minimal agar medium supplemented with either D-glucose, cellobiose or lactose. Additional file 2: Table S1. Chromosomal location of mutated genes in QM9136. Additional file 3: Figure S2. cDNA alignment of native and mutated *xyr1* loci. cDNA alignment of the 5′-region of native *xyr1* and mutated *xyr1* ^*QM9136*^ loci showing the A2294 point deletion causing the frame-shift that introduces six stop codons; the first of which terminates translation pre-maturely leading to the truncated XYR1^1–780^. Additional file 4: Figure S3. Alignment of the C-terminus of XYR1 with the orthologs from several Pezizomycota. Abbreviations (=gene bank entries) specify: AAV31053.1, *Penicillium canescens*; AAZ75672.1, *Sclerotinia sclerotiorum*; aor:AO090012000267, *Aspergillus oryzae*; BAB62022.1, *A. kawachii*; EAA33375.1, *Neurospora crassa;* EAQ87362.1, *Chaetomium globosum*; XP_363488.1, *Magnaporthe grisea*; Xyr1_9136, *T. reesei* QM9136; XYR1_WT, *T. reesei* QM6a (parental strain). Additional file 5: Figure S4. Detection of GFP-XYR1 under autofluorescence background eliminating conditions. To verify that the generally weak GFP-XYR1^1–780^ fluorescence signals were no artifacts, microscope settings were optimized to eliminate cellular background auto-fluorescence, so that any detectable fluorescence signal must emanate from a GFP source. For this test we deliberately choose a weak inducer (1 mM D-xylose for 30 min) to evaluate GFP-XYR1^1–780^ fluorescence at the lower end of the protein expression and microscope detection range, respectively. **(A)** The truncated GFP-XYR1^1–780^ protein was detectable with an average intensity of about 4.5-times less compared to the full-length GFP-XYR1 construct. Interestingly, under these conditions GFP-XYR1^1–780^ was localized as small cellular clusters (spots), and its cytoplasmic fraction was slightly elevated. This possibly suggests less efficient nuclear import due to proteasomal degradation of the non-functional construct. Notably, as shown above, in the presence of stronger cellulase-inducing carbon sources GFP-XYR1^1–780^ does - although highly inefficient - accumulate inside nuclei. Nevertheless, in conclusion, this experiment confirmed that the truncated XYR1 allele becomes expressed and responds to cellulase-induction, however, its production within 30 minutes on 1 mM D-xylose medium cannot be upregulated sufficiently, therefore leading to a low signal-to-noise ratio. (n.d. = not detectable). **(B)** Representative fluorescence and corresponding bright field images of the experimental conditions quantified in **(A)**. Additional file 6: Figure S5.
*Xyr1* gene replacement cassette. Schematic representation of the replacement cassette for exchanging the native *xyr1* locus in QM9136 and QM9414, respectively, with a *gfp-xyr1* encoding fragment by homologous recombination. The approximate position of the A2294 point deletion inserted in the *gfp-xyr1* ^∆*A2294*^ construct, leading to the expression of the truncated GFP-XYR1^1–780^ protein, is indicated by an asterisk.

## Abbreviations

AAD: Acidic activation domain
ATCC: American type culture collection
BLAST: Basic local alignment search tool
CBH1: Cellobiohydrolase 1
CDR: Coding region
DBD: DNA-binding domain
FSTFD: Fungal-specific transcription factor domain
FTFAD: Fungal transcription factor activation domain
FTFRMH: Fungal transcription factor regulating middle homology (domain)
GAI: Genome analyser 1
GFP: Green fluorescent protein
indels: Insertions and deletions
JGI: Joint Genome Institute
MA: Mandels-Andreotti
NADH: nicotinamid adenine dinucleotide (reduced form)
NLS: Nuclear localization signal
PDA: Potato dextrose agar
QM: Quater master (strain collection)
RUT: Rutgers (mutagenesis program)
SNP: Short nucleotide polymorphism
SNV: Short nucleotide variants
SRA: Sequence read archive
TF: Transcription factor
TRAL: *Trichoderma reesei* Alexander Lichius
Trire: *Trichoderma reesei*
US: United States
WWII: Second world war
XYN2: Xylanase 2
XYR1: Xylanase regulator 1
