# Erratum to: Genome sequencing of the *Trichoderma reesei* QM9136 mutant identifies a truncation of the transcriptional regulator XYR1 as the cause for its cellulase-negative phenotype

**DOI:** 10.1186/s12864-015-1917-2

**Published:** 2015-09-22

**Authors:** Alexander Lichius, Frédérique Bidard, Franziska Buchholz, Stéphane Le Crom, Joel Martin, Wendy Schackwitz, Tina Austerlitz, Igor V. Grigoriev, Scott E. Baker, Antoine Margeot, Bernhard Seiboth, Christian P. Kubicek

**Affiliations:** Research Division Biotechnology and Microbiology, Institute of Chemical Engineering, Vienna University of Technology, A-1060 Vienna, Austria; IFP Energies Nouvelles, 1-4 Avenue de Bois-Préau, 92852 Rueil-Malmaison, France; Sorbonne Universités, UPMC Université Paris 06, Institut de Biologie Paris Seine (IBPS), FR 3631, Département des Plateforme, F-75005 Paris, France; US Department of Energy Joint Genome Institute, 2800 Mitchell Avenue, Walnut Creek, CA 94598 USA; Environmental Molecular Sciences Laboratory, Pacific Northwest National Laboratory, Richland, WA 99354 USA

Erratum to: BMC Genomics doi 10.1186/s12864-015-1526-0

Following the publication of our recent article in *BMC Genomics* [1] we wish to bring the following corrigendum to your attention. In the above paper, we wrote in the discussion on page 13: “These are V756F in XlnR (corresponding to V801 in XYR1) and A804V (based on our analysis; not A824V as stated by the authors) in XYR1”. Our analysis of the XYR1 sequence was based on the available *Trichoderma reesei* QM6a XYR1 sequences deposited in the NCBI database [Protein Accession Number XP_006966092.1 and EGR48040.1]. Recently, Derntl *et al*. [2] identified that the second intron in *xyr1* is in fact not spliced thus giving rise to a protein that, while maintaining the reading frame, is 20 amino acids longer. Consequently, the position A824V given by Derntl *et al.* [2] is correct, and the numbering of amino acids in our paper after G319 has to be increased by 20.

A corrected version of our statement would read: "These are V756F in XlnR of *A. niger* which corresponds to V821 in *T. reesei* XYR1, as well as A824V in XYR1 of *T. reesei* [35, 42]".

We apologize for this mistake, but would like to stress that none of the results or conclusions in our paper are affected by this change.
