# Supplementary figures and images for: Genome sequencing of the Trichoderma reesei QM9136 mutant identifies a truncation of the transcriptional regulator XYR1 as the cause for its cellulase-negative phenotype
Source: BMC Genomics. 2015 Apr 20;16(1):326. doi: 10.1186/s12864-015-1526-0 (PMC4409711; doi:10.1186/s12864-015-1526-0)

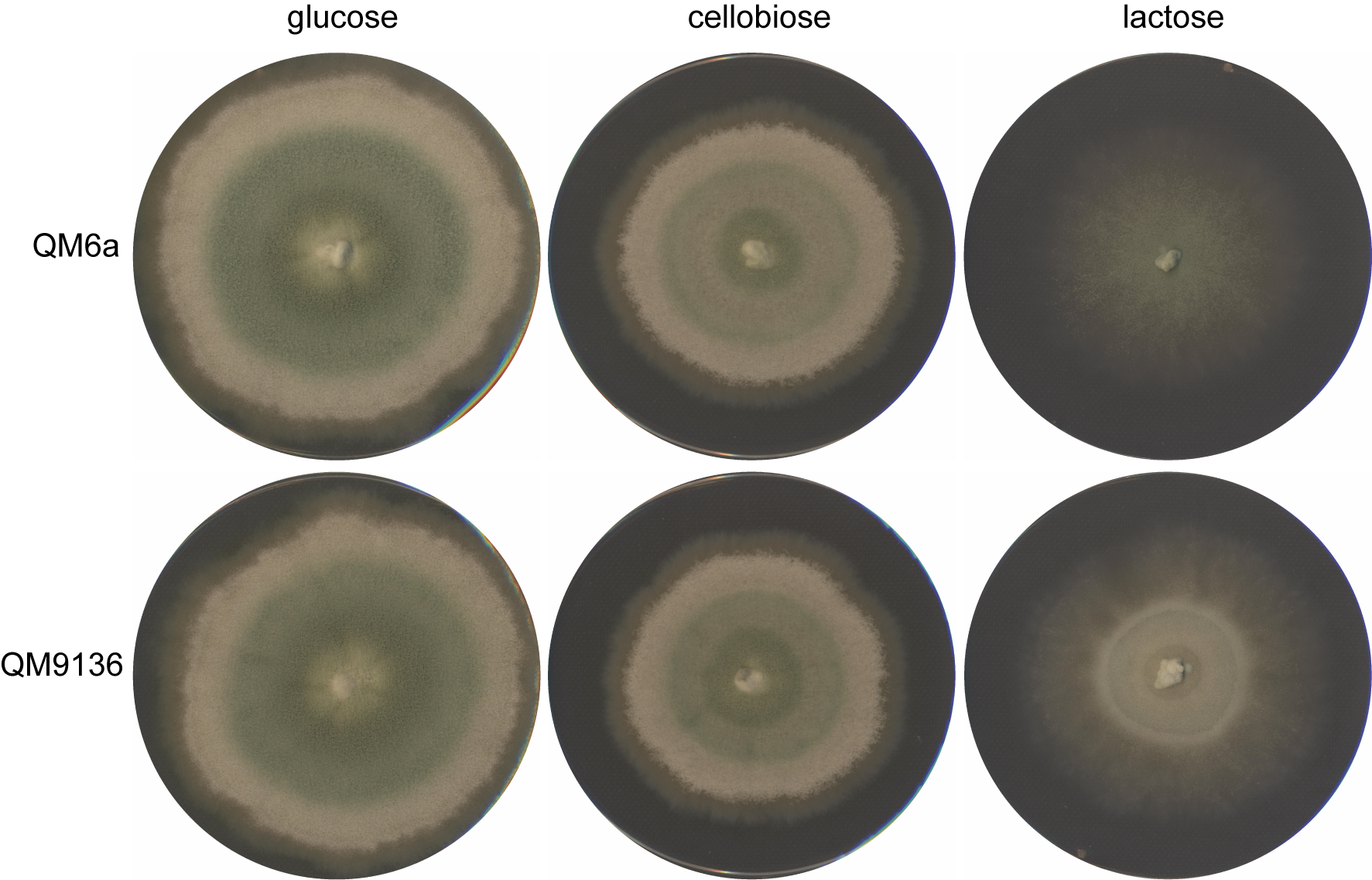

Supplement: Additional file 1: Figure S1. — Phenotypic comparison of QM6a and QM9136. Growth of T. reesei QM6a (parental strain) and QM9136 on solid minimal agar medium supplemented with either D-glucose, cellobiose or lactose. [file 12864_2015_1526_MOESM1_ESM.tiff]

**Figure S3 new**


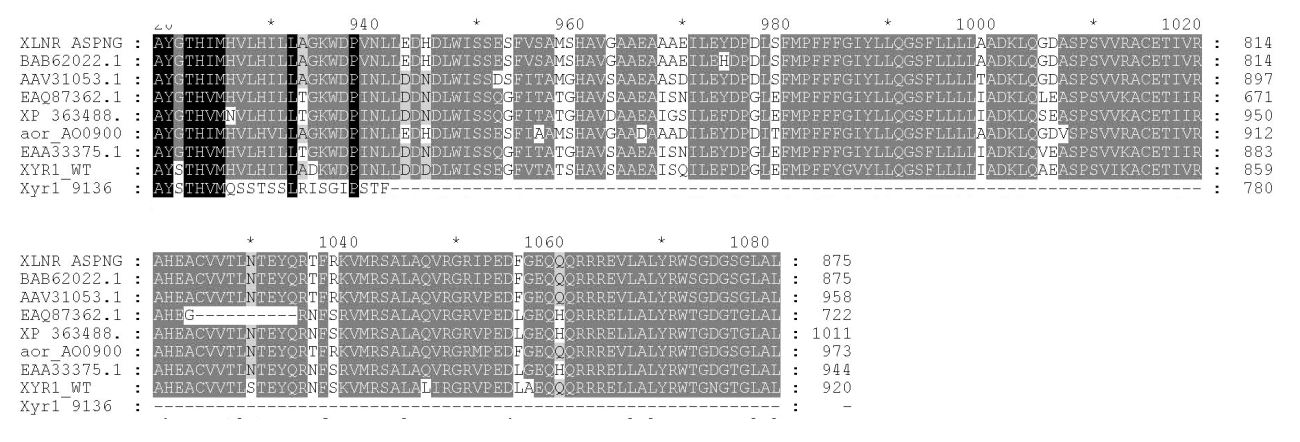

Supplement: Additional file 4: Figure S3. — Alignment of the C-terminus of XYR1 with the orthologs from several Pezizomycota. Abbreviations (=gene bank entries) specify: AAV31053.1, Penicillium canescens; AAZ75672.1, Sclerotinia sclerotiorum; aor:AO090012000267, Aspergillus oryzae; BAB62022.1, A. kawachii; EAA33375.1, Neurospora crassa; EAQ87362.1, Chaetomium globosum; XP_363488.1, Magnaporthe grisea; Xyr1_9136, T. reesei QM9136; XYR1_WT, T. reesei QM6a (parental strain). [file 12864_2015_1526_MOESM4_ESM.docx]

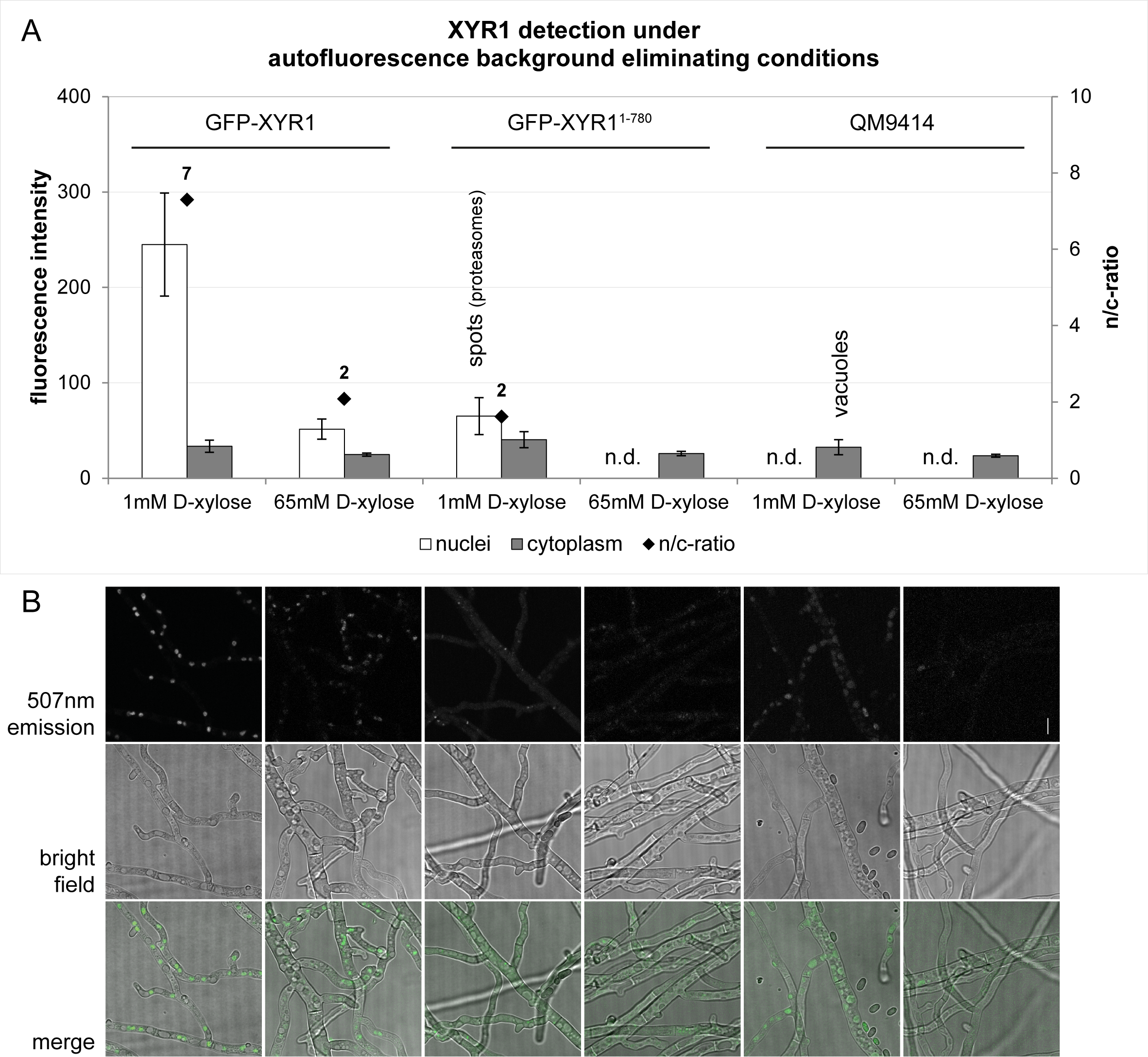

Supplement: Additional file 5: Figure S4. — Detection of GFP-XYR1 under autofluorescence background eliminating conditions. To verify that the generally weak GFP-XYR11–780 fluorescence signals were no artifacts, microscope settings were optimized to eliminate cellular background auto-fluorescence, so that any detectable fluorescence signal must emanate from a GFP source. For this test we deliberately choose a weak inducer (1 mM D-xylose for 30 min) to evaluate GFP-XYR11–780 fluorescence at the lower end of the protein expression and microscope detection range, respectively. (A) The truncated GFP-XYR11–780 protein was detectable with an average intensity of about 4.5-times less compared to the full-length GFP-XYR1 construct. Interestingly, under these conditions GFP-XYR11–780 was localized as small cellular clusters (spots), and its cytoplasmic fraction was slightly elevated. This possibly suggests less efficient nuclear import due to proteasomal degradation of the non-functional construct. Notably, as shown above, in the presence of stronger cellulase-inducing carbon sources GFP-XYR11–780 does - although highly inefficient - accumulate inside nuclei. Nevertheless, in conclusion, this experiment confirmed that the truncated XYR1 allele becomes expressed and responds to cellulase-induction, however, its production within 30 minutes on 1 mM D-xylose medium cannot be upregulated sufficiently, therefore leading to a low signal-to-noise ratio. (n.d. = not detectable). (B) Representative fluorescence and corresponding bright field images of the experimental conditions quantified in (A). [file 12864_2015_1526_MOESM5_ESM.tiff]

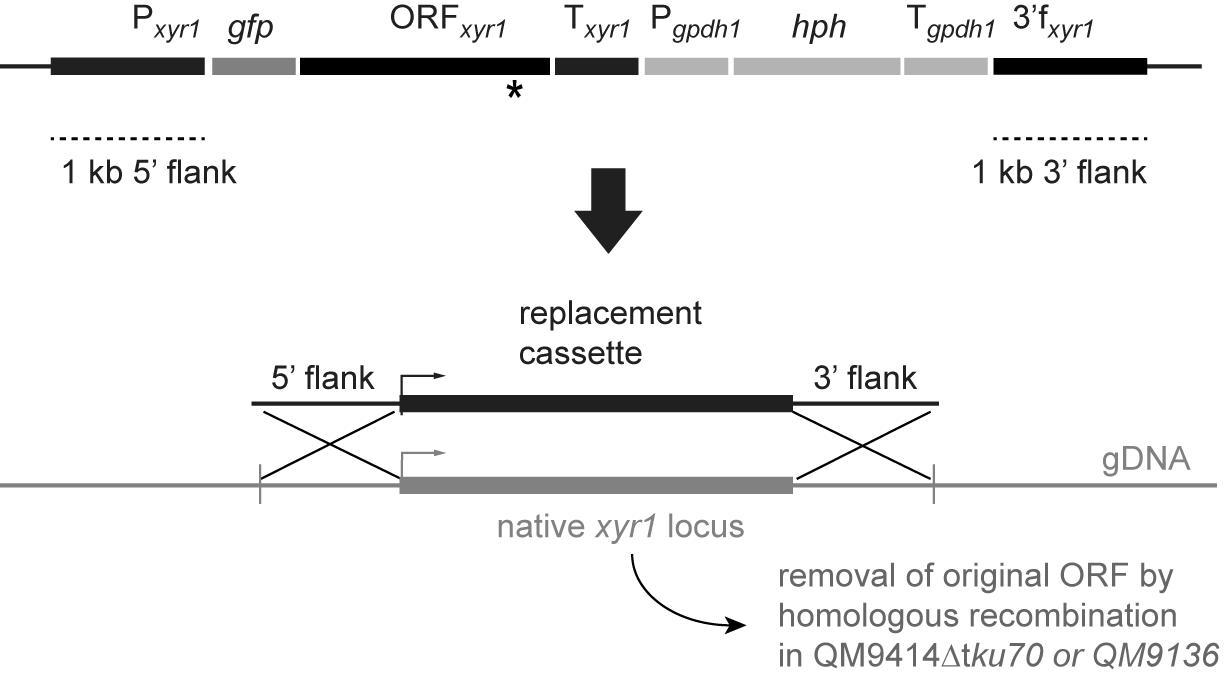

Supplement: Additional file 6: Figure S5. — Xyr1 gene replacement cassette. Schematic representation of the replacement cassette for exchanging the native xyr1 locus in QM9136 and QM9414, respectively, with a gfp-xyr1 encoding fragment by homologous recombination. The approximate position of the A2294 point deletion inserted in the gfp-xyr1 ∆A2294 construct, leading to the expression of the truncated GFP-XYR11–780 protein, is indicated by an asterisk. [file 12864_2015_1526_MOESM6_ESM.tiff]
