# Supplementary material for: Genome sequencing of the Trichoderma reesei QM9136 mutant identifies a truncation of the transcriptional regulator XYR1 as the cause for its cellulase-negative phenotype
Source: BMC Genomics. 2015 Apr 20;16(1):326. doi: 10.1186/s12864-015-1526-0 (PMC4409711; doi:10.1186/s12864-015-1526-0)
Supplement: Additional file 2: Table S1. — Chromosomal location of mutated genes in QM9136. [file 12864_2015_1526_MOESM2_ESM.doc]

**Table S1.** Chromosomal location of mutated genes in QM9136.

| **scaffold** | **scaffold size** | **Trire2:** | | **gene ORF** | |
| --- | --- | --- | --- | --- | --- |
|  | **[bp]** |  | **begin** | | **end** |
| 1 | 2756989 | 102500 | 518168 | | 518761 |
| 4 | 1832615 | 46238 | 1495404 | | 1496693 |
| 11 | 1155933 | 122208 | 201774 | | 204723 |
| 15 | 837556 | 109416 | 823530 | | 831870 |
| 18 | 685578 | 50707 | 606893 | | 608754 |
| 19 | 663018 | 66687 | 226345 | | 227457 |
| 23 | 512080 | 5387 | 109676 | | 110866 |
| 31 | 231272 | 124043 | 172323 | | 173903 |
