# Supplementary material for: Genome sequencing of the Trichoderma reesei QM9136 mutant identifies a truncation of the transcriptional regulator XYR1 as the cause for its cellulase-negative phenotype
Source: BMC Genomics. 2015 Apr 20;16(1):326. doi: 10.1186/s12864-015-1526-0 (PMC4409711; doi:10.1186/s12864-015-1526-0)
Supplement: Additional file 3: Figure S2. — cDNA alignment of native and mutated xyr1 loci. cDNA alignment of the 5′-region of native xyr1 and mutated xyr1 QM9136 loci showing the A2294 point deletion causing the frame-shift that introduces six stop codons; the first of which terminates translation pre-maturely leading to the truncated XYR11–780. [file 12864_2015_1526_MOESM3_ESM.docx]

**Figure S2 new**

xyr1 AACGCGTCCAGCCGCATGACGGAGAGCGAGATCCAGGCCAGCATCGTGGTGGCTTACAGC 2280

xyr1_QM9136 AACGCGTCCAGCCGCATGACGGAGAGCGAGATCCAGGCCAGCATCGTGGTGGCTTACAGC 2280

************************************************************

xyr1 ACCCATGTGATGCATGTCCTCCACATCCTCCTTGCGGATAAGTGGGATCCCATCAACCTT 2340

xyr1_QM9136 ACCCATGTGATGC-TGTCCTCCACATCCTCCTTGCGGATAAGTGGGATCCCATCAACCTT 2339

************* **********************************************

deleted A2294

xyr1 CTAGACGACGACGACTTGTGGATCTCGTCGGAAGGATTCGTGACGGCGACGAGCCACGCG 2400

xyr1_QM9136 CTAGGACGACGACGACTTGTGGATCTCGTCGGAAGATTCGTGACGGCGACGAGCCACGCG 2399

************************************************************

premature stop codon

xyr1 GTATCGGCTGCCGAAGCTATTAGCCAGATTCTCGAGTTTGACCCTGGCCTGGAGTTTATG 2460

xyr1_QM9136 GTATCGGCTGCCGAAGCTATTAGCCAGATTCTCGAGTTTGACCCTGGCCTGGAGTTTATG 2459

************************************************************

xyr1 CCATTCTTCTACGGCGTCTATCTCCTGCAGGGTTCCTTCCTCCTCCTGCTCATCGCCGAC 2520

xyr1_QM9136 CCATTCTTCTACGGCGTCTATCTCCTGCAGGGTTCCTTCCTCCTCCTGCTCATCGCCGAC 2519

************************************************************

xyr1 AAGCTGCAGGCCGAAGCGTCTCCAAGCGTCATCAAGGCTTGCGAGACCATTGTTAGGGCA 2580

xyr1_QM9136 AAGCTGCAGGCCGAAGCGTCTCCAAGCGTCATCAAGGCTTGCGAGACCATTGTTAGGGCA 2579

************************************************************

xyr1 CACGAAGCTTGCGTTGTGACGCTGAGCACAGAGTATCAGCGCAACTTTAGCAAGGTTATG 2640

xyr1_QM9136 CACGAAGCTTGCGTTGTGACGCTGAGCACAGAGTATCAGCGCAACTTTAGCAAGGTTATG 2639

************************************************************

xyr1 CGAAGCGCGCTGGCTCTGATTCGGGGCCGTGTGCCGGAAGATTTAGCTGAGCAGCAGCAG 2700

xyr1_QM9136 CGAAGCGCGCTGGCTCTGATTCGGGGCCGTGTGCCGGAAGATTTAGCTGAGCAGCAGCAG 2699

************************************************************

xyr1 CGACGACGCGAGCTTCTTGCACTATACCGATGGACTGGTAACGGAACCGGTCTGGCCCTC 2760

xyr1_QM9136 CGACGACGCGAGCTTCTTGCACTATACCGATGGACTGGTAACGGAACCGGTCTGGCCCTC 2759

************************************************************

xyr1 TAA 2763

xyr1_QM9136 TAA 2762
